# Genome-wide characterization and analysis of bHLH transcription factors related to anthocyanin biosynthesis in spine grapes (*Vitis davidii*)

**DOI:** 10.1038/s41598-021-85754-w

**Published:** 2021-03-25

**Authors:** Ming Li, Lei Sun, Hong Gu, Dawei Cheng, XiZhi Guo, Rui Chen, Zhiyong Wu, Jianfu Jiang, Xiucai Fan, Jinyong Chen

**Affiliations:** 1 Zhengzhou Fruit Research Institute, Chinese Academy of Agricultural Sciences, Zhengzhou, 450009 China; 2 Biotechnology Research Institute, Tianjin Academy of Agricultural Sciences, Tianjin, 300192 China

**Keywords:** Molecular biology, Plant sciences

## Abstract

As one of the largest transcription factor family, basic helix-loop-helix (bHLH) transcription factor family plays an important role in plant metabolism, physiology and growth. Berry color is one of the important factors that determine grape quality. However, the bHLH transcription factor family’s function in anthocyanin synthesis of grape berry has not been studied systematically. We identified 115 bHLH transcription factors in grape genome and phylogenetic analysis indicated that bHLH family could be classified into 25 subfamilies. First, we screened six candidate genes by bioinformatics analysis and expression analysis. We found one of the candidate genes *VdbHLH037* belonged to III (f) subfamily and interacted with genes related to anthocyanin synthesis through phylogenetic analysis and interaction network prediction. Therefore, we speculated that *VdbHLH037* participated in the anthocyanin synthesis process. To confirm this, we transiently expressed *VdbHLH037* in grape and *Arabidopsis* transformation. Compared with the control, transgenic materials can accumulate more anthocyanins. These results provide a good base to study the function of the VdbHLH family in anthocyanin synthesis of grape berry.

## Introduction

Grapes (*Vitis vinifera L.*) are one of the most economically valuable fruit crops in the world, mainly used in winemaking, fresh food and fruit juice^1^. However, the appearance of grapes is affected by uneven coloration. With the continuous improvement in people’s living standards, the requirements for grape appearance are higher. Therefore, berry color is a key factor in determining the commerciality of grape products. Anthocyanins play a vital role in the coloring process of grapes. There are more than 250 kinds of anthocyanins with known structures in plants that are classified into six main categories: cyanidin, delphinidin, pelargonidin, peonidin, petunidin and malvidin^2^. The grape skin is rich in anthocyanins, and the composition and content of anthocyanins generate the different berry colors. For example, petunidin and malvidin give the berry a mazarine and purple color, and the cyanidin and pelargonidin make the berry bright red. Other factors such as co-pigmentation and pH also affect the berry color^3^. In addition, anthocyanins play an important role in response to biotic and abiotic stresses. Castellarin et al. found that water stress induced change in gene expression regulating flavonoid biosynthesis in grape berries^4^. Gutha et al. found that compared with normal grape leaves, the leaves infected with leaf roll virus accumulated more anthocyanin, and the expression level of genes involved in flavonoid synthesis pathway was also significantly increased^5^.

Anthocyanins are important secondary metabolites belonging to the flavonoid compound that are synthesized by the condensation of anthocyanins and glycoside via glycoside bonds. They are widely found in roots, stems, flowers and fruits of plants. The anthocyanin biosynthesis pathway has more than 20 steps catalyzed by a series of enzymes. Its synthesis is regulated by structural and regulatory genes. Structural genes mainly include *CHS* (*chalcone synthase*), *CHI* (*chalcone isomerase*), *F3H* (*flavanone3-hydroxylase*), *DFR* (*dihydroflavonol4-reductase*), *ANS* (*anthocyanidin synthase*) and *UFGT* (*flavonoid3-O-glucosyltransferase*)^6^. These structural genes are regulated, in many plants, by MBW regulatory complex composed of R2R3 MYB TFs, bHLH protein and WD40-repeat protein^7^. Studies have shown that MYB is one of the most important transcription factors regulating anthocyanin biosynthesis^8^. The first MYB transcription factor in plants was found in maize, and was involved in the metabolic regulation of flavonoids^9^. MYB is the most widely distributed transcription factor family, with 138 members in grapes. It was found that *MybA* regulated anthocyanin synthesis through the expression of UFGT in Kyoho grape^10^. Kobayashi et al. found that white coloration is caused by retrotransposon insertion in *MybA* and that the same mutant allele has spread among most white grape cultivars in the world^11^. bHLH is also a major transcription factor regulating anthocyanin biosynthesis, which can directly activate DFR expression and can also promote anthocyanin accumulation by interacting with MYB. The WD40 transcription factor associated with coloration was also found in grapes. It was functionally verified and found to be expressed only on colored grapes^12^.

The bHLH domain consists of 50–60 amino acids that can be divided into basic region and HLH region^13^.The basic region at the N-terminus that contains approximately 15 amino acids is responsible for binding to DNA^14^. It has a highly conserved HER motif (His5-Glu9-Arg3) that recognizes a consensus hexanucleotide sequence E-box. Based on the different central bases, E-box is of different types, of which G-box is the most common type. Two amphipathic α-helices of the HLH region consisting of roughly 40 amino acids are separated by a loop of variable length^15^. It can promote the formation of homodimers or heterodimers through interacting with other bHLH proteins^16^.

The bHLH protein family is one of the largest families of TFs in plants; for example, there are 162 *bHLH* genes in *Arabidopsis thaliana*^17^, 192 in tobacco (Nicotiana tabacum)^18^, 159 in tomato (*Solanum lycopersicum*)^19^, 188 in apple (*Malus* × *domestica*)^20^, and 113 in strawberry (*Fragaria* × *ananassa*)^21^. It is usually divided into 15–26 subfamilies. For instance, 162 bHLH members of *Arabidopsis* are classified into 26 subfamilies. 230 bHLH proteins in Chinese cabbage (*Brassica rapa ssp. pekinensis*) are divided into 24 subfamilies^13^. Apple contains 18 subfamilies according to the phylogenetic tree analysis^20^. In plants, transcription factors belonging to the same subfamily have high similarity in structure, motif and protein function^22^. bHLH family are involved in many developmental and physiological processes in plants, such as anthocyanin biosynthesis^23,24^, growth and development^25,26^, and defense response to biotic and abiotic stress^27,28^. Different types of *bHLH* genes have different biological functions. It has been shown that bHLH III (d + e) subfamily members can enhance plant resistance and regulate anthocyanin synthesis through JA signaling pathway^29,30^. In addition, members of the III (f) subfamily have been proved to be involved in anthocyanin synthesis^31^.

The berry color of Chinese wild grapes is generally black, the spine grape has both black and white berry, which is of great significance for studying the coloring mechanism of grape berry. In this study, we analyzed 115 bHLH transcription factors comprehensively and systematically in spine grape. First, we mapped the bHLH transcription factors on to the 19 chromosomes of grape. Then we analyzed phylogenetic relationships, gene structural features, distribution of key amino acid residues in bHLH domain, conserved motifs and protein interaction network of bHLH transcription factors. Finally, six candidate genes were screened by combining with results of transcriptome sequencing and qRT-PCR validation. We verified *VdbHLH037* of III (f) subfamily function by heterologous expression in *Arabidopsis thaliana* and transient expression in “Kyoho” grapes. The results show that *VdbHLH037* plays a positive role in the regulation of anthocyanin synthesis.

## Results

### Identification and annotation of bHLH transcription factors in grape

**Figure 1:**
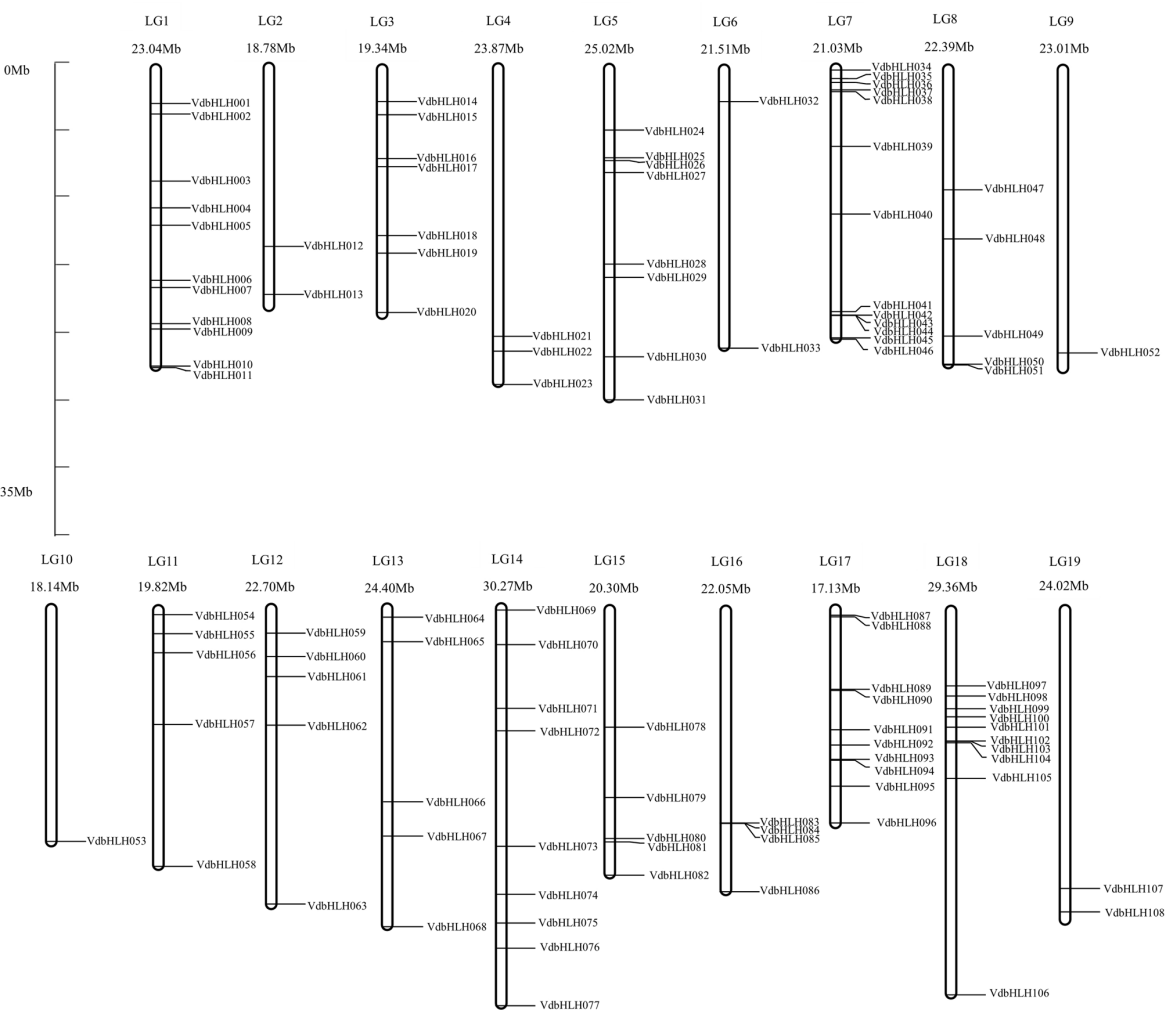
The distribution of 115 *VdbHLH* genes on the nineteen grape chromosomes. The centromeric positions are shown according to the location of each *VdbHLH* gene.

A total of 137 bHLH transcription factors were obtained from *EnsemblPlants* database. Subsequently, we used the conserved domain to determine the existence of the conserved bHLH domain. Some members without the bHLH domain were removed. Then we analyzed the nucleotide and amino acid sequences through SMART and DNAStar. If the sequences were similar, only one member with the longest sequence was left. Finally, we screened 115 *VdbHLH* members for the further analysis according to the results of transcriptome sequencing^15^. We collated the information of 115 *VdbHLH* transcription factors from the Plant Transcription Factor Database (Supplementary Table S1). We mapped the *VdbHLH* transcription factors on the 19 chromosomes according to their distributions on the chromosome (Fig. 1). Then we renamed them from *VdbHLH001* to *VdbHLH108* based on their location on the chromosome 1–19 from the Plant Transcription Factor Database. The remaining six unknown chromosomal loci were renamed from *VdbHLH109* to *VdbHLH115* in the order of position from the minimum to maximum. We will further study the biological functions of 115 *VdbHLH* genes, especially for anthocyanin synthesis.

### Phylogenetic analysis of the bHLH transcription factor family in grape

**Figure 2:**
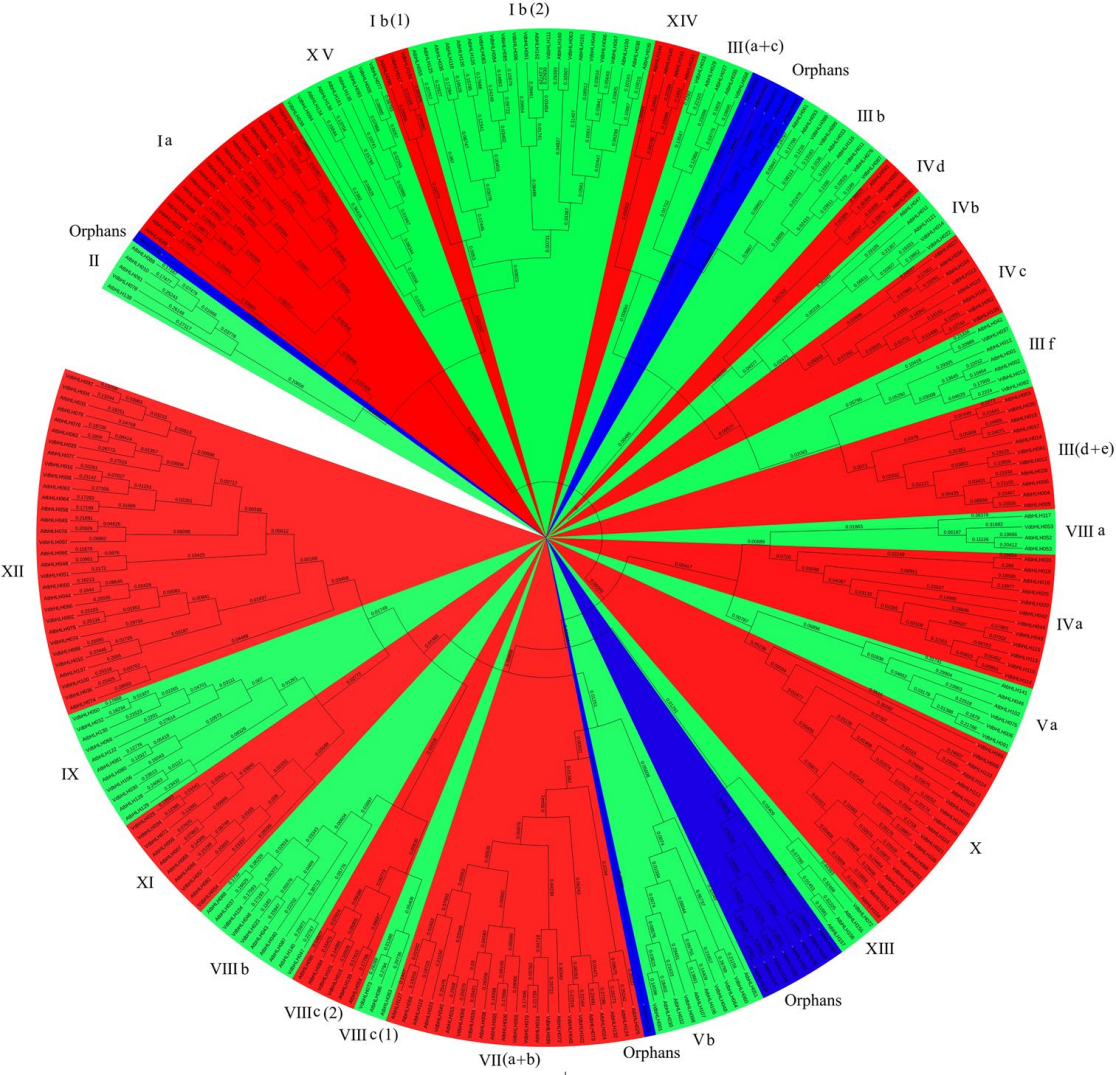
An un-rooted phylogenetic tree of the VdbHLH family was constructed through the neighbor-joining method. The phylogenetic tree was constructed online with Interactive Tree of Life (iTOL). The numbers are bootstrap values based on 1000 iterations. Only bootstrap values larger than 50% support are displayed.

In order to better understand the function of grape bHLH protein, we need to assess their evolutionary relationship. Based on 162 AtbHLHs in *Arabidopsis*, we constructed an un-rooted phylogenetic tree with the nomenclature protocol according to the multiple sequence alignments of the conserved domains in grape and *Arabidopsis*^31^ (Fig. 2). On the basis of Heim’s method, we made appropriate adjustments. For example, I (a + b) subfamily was divided into I a, I b(1) and I b(2) subfamilies. III a subfamily and III c subfamily were merged into III (a + c) subfamily. Ultimately, the grape bHLH family was divided into 25 subfamilies. The members that were not assigned to the 25 subfamilies were classified as “orphans”. The plant bHLH family consists of approximately 26 subfamilies^32,33^, whereas there is no bHLH member in the grape X IV subfamily, probably due to the long evolutionary process. We found that the number of members of different subfamilies varies greatly. For example, the largest subfamily X II contains 14 members, while the smallest subfamilies II, VIII a, VIII c(1) and X III contain only one member. The classification of the grape bHLH family provides evidence of their evolutionary relationship.

### Gene structure and conserved motif analysis of VdbHLH genes

In order to obtain information about the *VdbHLH* family, we analyzed the structural characteristics of the *VdbHLH* family and labeled the phase information through GSDS2.0 (Gene Structure Display Server) (Supplementary Fig. S1). The results of the structural analysis showed that the number of exons ranged from 1 to 11. Four members had no introns. The average number of exons in 25 subfamilies ranged from 1 (VIII b) to 9 (V a). All four members without introns belong to the (VIII.

b) family. Introns can occur anywhere in the gene. If the intron is located between the third nucleotide of one codon and the first nucleotide of another codon, it is called phase 0 intron. Correspondingly, the introns between the first nucleotide and the second nucleotide of a codon are called the phase 1 introns. The introns between the second nucleotide and the third nucleotide of a codon are called the phase 2 introns. This is very important for the process of exon replication. Exons between two identical phases are called symmetric exons, whose nucleotide number is an integer multiple of three, which will not cause the reading frame to be shifted and can be copied. In contrast, asymmetric exons cannot be replicated. It is generally believed that symmetric exons and phase 0 introns can facilitate exon shuffling, recombinational fusion and protein domain exchange^34,35^. The statistics of 601 exons are as follows: 53 symmetrical exons between phase 0 introns, 37 symmetrical exons between phase1 intron and 52 symmetrical exons between phase 2 introns. Among the 646 introns, 190 are phase 0 introns, 133 are phase 1 introns and 323 are phase 2 introns. The results of these analyses illustrate the diversity of the *VdbHLH* family.

It is generally believed that motifs play an important role in the interaction of different modules in transcriptional complexes and transduction of signal^15,31^. In addition, the structure of a motif is closely related to gene classification. Therefore, we analyzed the 20 conservative motifs of the *VdbHLH* family and their distribution among different subfamilies using MEME (Supplementary Fig. S2). Furthermore, we also counted the sequence, length and number of occurrences of motifs (Supplementary Table S2). The subfamily IV a has the most types of motifs (nine types) and subfamily X III has the least types of motif (one type). The average number of motifs ranges from two (II, VIII a, VIII b, VIII c and X V) to seven (III f). All motifs occur only once per gene. Some motifs are universal, 113 members have motif2, and 96 members have motif1. Some motifs are conservative and only appear in a particular subfamily. For example: motif19 exists only in subfamily I B (2) (*VdbHLH049*, *VdbHLH066* and *VdbHLH067*), motif20 exists only in subfamily III b (*VdbHLH011*, *VdbHLH076* and *VdbHLH087*) and motif18 exists only in subfamily III f (*VdbHLH013*, *VdbHLH037* and *VdbHLH082*). This may be why each subfamily has a specific biological function. The analysis of motif and gene structure provides a further theoretical basis for the *VdbHLH* subfamily classification.

### The conserved amino acid residues in bHLH domain and their ability of DNA-binding

**Figure 3:**
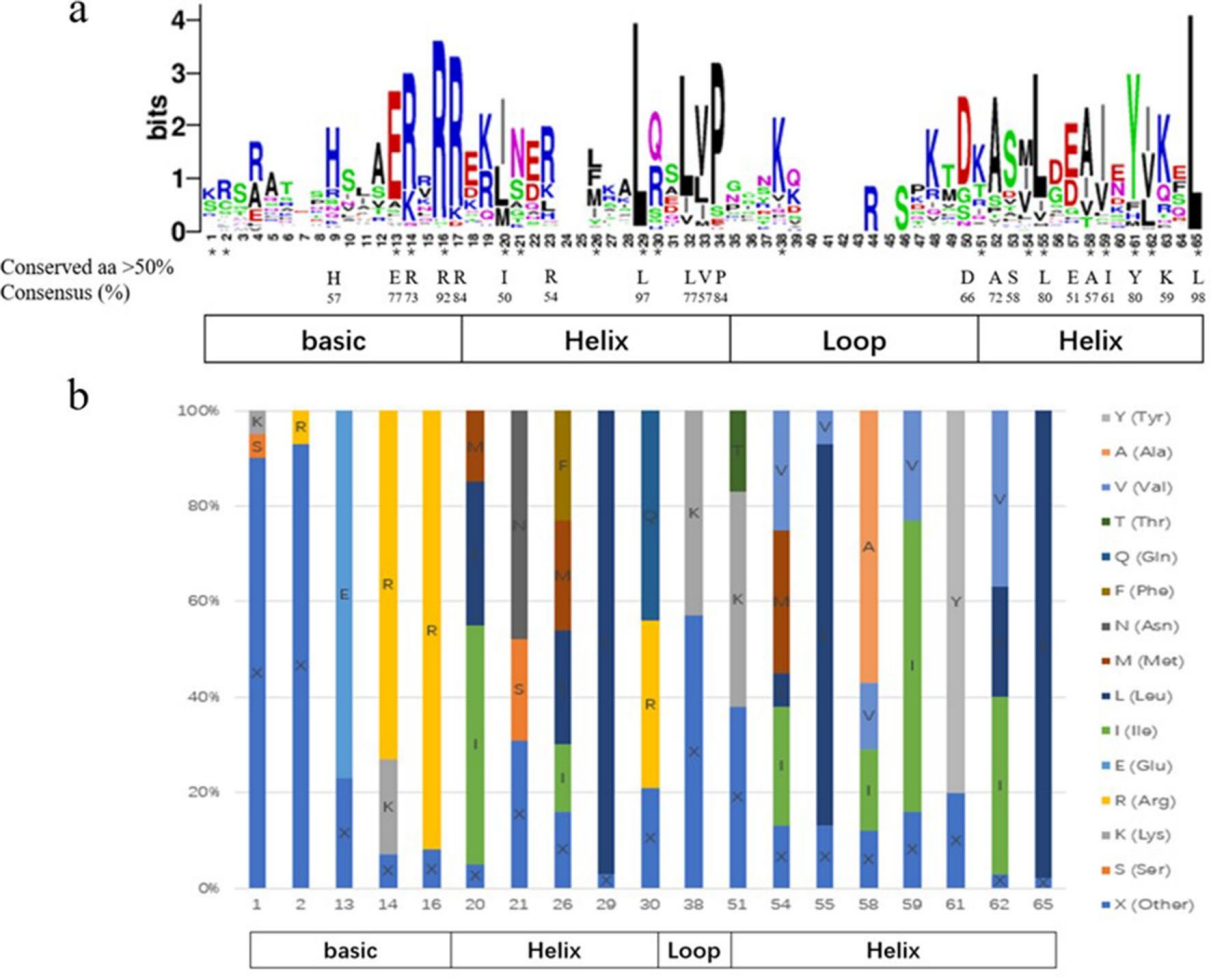
The characterization and distribution of bHLH domains across all VdbHLH. **(a)** The sequence logo of the bHLH domain. The asterisks represent the 19 conserved amino acids mentioned previously by Atchley. The capital letters represent conserved amino acids that contain more than 50%. (**b**) The distribution of amino acids in the bHLH domain. The columns of different colors represent the percentage of amino acids at this site.

To further understand the function of the *VdbHLH* family, we performed multiple sequence alignment of 115 VdbHLH domains and calculated the percentage of conserved amino acids based on previous results (Fig. 3). The bHLH domain is made up of one basic region, two helix regions and one loop region. The results show that the conservation of basic region and helix region is higher than that of the loop region. The bHLH region of grape is composed of 65 amino acid residues, of which 21 conserved amino acid residues with a consensus rate of more than 50%. Among them, Arg-16, Leu-29 and Leu-65 are extremely conservative, with a consensus rate of more than 90% (Supplementary Table S3). Previous studies have shown that Glu-13, Arg-16 and Arg-17 in the basic region are critical for DNA binding, and Leu-29 and Leu-65 in the helix region are very important for dimerization activity^26^. By comparison, we found that seven amino acid residues (Ile-20, Leu-26, Gln-30, Met-54, Ile-59, Ile-62 and Leu-65) in plants are more conserved than those in animals, indicating that they play a very important role in plants.

It is generally believed that the basic region is critical for DNA binding and the bHLH family to achieve its biological function^36^. According to Atchley’s research, the basic region of the bHLH protein has 13 amino acid residues, whereas the grape contains 17 amino acid residues, of which more than five are responsible for DNA binding^37^. The number of amino acid residues in the basic region of *VdbHLH089* is less than six, which is considered incapable of DNA binding, so we designated it as a non-DNA binding protein^19^. As previously reported, we have divided the remaining 114 VdbHLH members into three categories: G-box (His/Lys-9, Glu-13 and Arg-17), E-box (Glu-13 and Arg-16) and non-E-box (Glu-13 and Arg-16 do not appear together)^38,39^. Based on the conserved amino acid residues in the basic region, we predicted 60 for the G-box-binding proteins, 27 for the non-G-box-binding proteins and 27 for the non-E-box-binding proteins. In addition, one of them was classified as a non-DNA-binding protein (Supplementary Table S4).

### The collinear correlations of bHLH genes in grape, Arabidopsis and tomato

Comparative genomics is a powerful tool for quickly understanding, localizing and cloning unknown genes by comparing genes and genomic structures. The rationale is that there is interspecific and intraspecific synteny. The synteny refers to the similarity in location and sequence of genes on interspecific and intraspecific homologous chromosomes^40^. The synteny analysis of the genome shows a large number of collinear regions in the genome that can be used as direct evidence for the existence of whole-genome duplication (WGD)^41,42^. Ohno first proposed the hypothesis of polyploidization, that is, genome-wide replication, to explain the way in which diploid genomes evolve into tetraploids through WGD^43^. In 1997, Wolfe et al. confirmed the WGD hypothesis in *Saccharomyces cerevisiae* genome^44^. In 2000, the first plant genome-wide sketch was sequenced and analyzed. The evolutionary history of *Arabidopsis* genome suggests that it has undergone two whole-genome duplications (WGD: α and β) and one whole-genome triplication (WGT: γ)^45^. In 2007, the genome of grapes was sequenced. Studies have shown that the ancestral genome of grapes may be paleohexaploid^46^. The study of grape genome duplication events laid the theoretical foundation for hexaploid sharing of the ancestral genomes of dicotyledons and opened doors for the study of the evolutionary history of ancestral genomes in angiosperms. Subsequently, the whole-genome sequencing of 14 fruit trees species was completed. The whole-genome sequencing results of these fruit tree species have established a huge resource platform for fruit tree molecular biology research that not only helps in understanding their genome structure and functions but also has important guiding significance for exploring the origin and evolution of fruit trees, locating and cloning important functional genes and accelerating molecular breeding.

**Figure 4:**
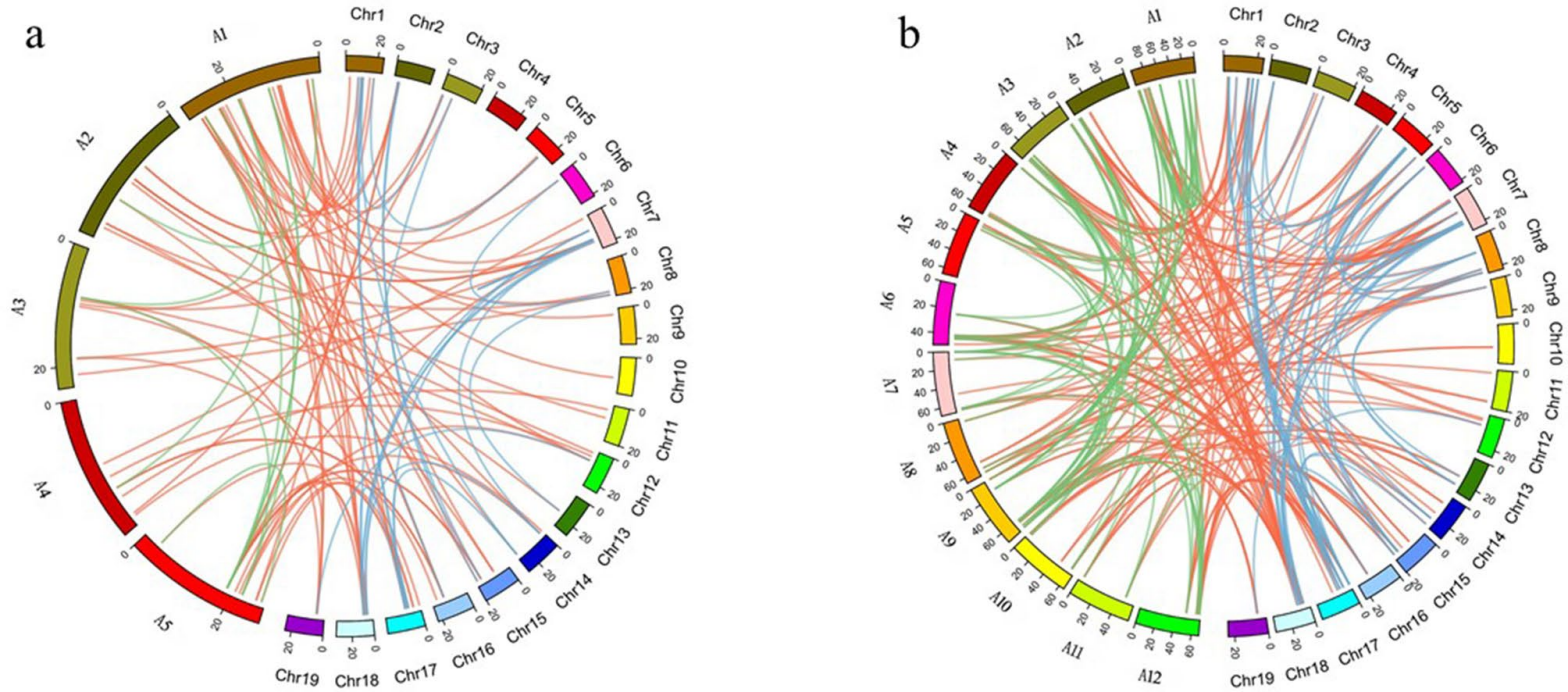
The analysis of paralogous *bHLH* genes and their orthologues in grape, *Arabidopsis* and tomato. (**a**) The analysis of paralogous and orthologues in grape (Chr1-Chr19) and *Arabidopsis* (A1-A5). The blue lines represent the paralogous *bHLH* genes in grape. The green lines represent the paralogous *bHLH* genes in *Arabidopsis*. The red lines represent their orthologous *bHLH* genes. (**b**) The analysis of paralogous and orthologues in grape (Chr1-Chr19) and tomato (A1-A12). The blue lines represent the paralogous *bHLH* genes in grape. The green lines represent the paralogous *bHLH* genes in tomato. The red lines represent their orthologous *bHLH* genes. The relationships of orthologous and paralogous *bHLH* genes among grape, *Arabidopsis* and tomato were drawn by Circos 0.69.6 (http://circos.ca/).

Comparative genomics confirmed that the grapes did not undergo any additional genome-wide replication event, while the ancestors in Solanaceae experienced an independent WGT event after differentiation^47^. Therefore, we carried out a few synteny analyses of grape and tomato genomes and found that the number of tomato genes was less than three times that of grapes. These results indicate that a large number of gene losses occur in WGD of tomato. We use OrthoMcl to analyze the orthologous *bHLH* genes in grape, *Arabidopsis* and tomato genome. We found 138 *bHLH* orthologous gene pairs between grape and tomato, but only 69 *bHLH* orthologous gene pairs between grape and *Arabidopsis* (Supplementary Table S5). The result was consistent with the close relationship between grape and tomato. In the analysis of synteny, we found each grape *bHLH* gene has one to three orthologous tomato genes. This shows that with the triplication of genome, some *bHLH* genes replicate as well. Through collinear correlations of the *bHLH* gene, we found 54, 10 and 75 paralogous *bHLH* gene pairs in grape, *Arabidopsis* and tomato, respectively (Supplementary Table S6). We used software Circos to show the relationships of orthologous and paralogous *bHLH* genes among grape, *Arabidopsis* and tomato (Fig. 4).

### The expression level of spine grape berry bHLH genes at different developmental stages

**Figure 5:**
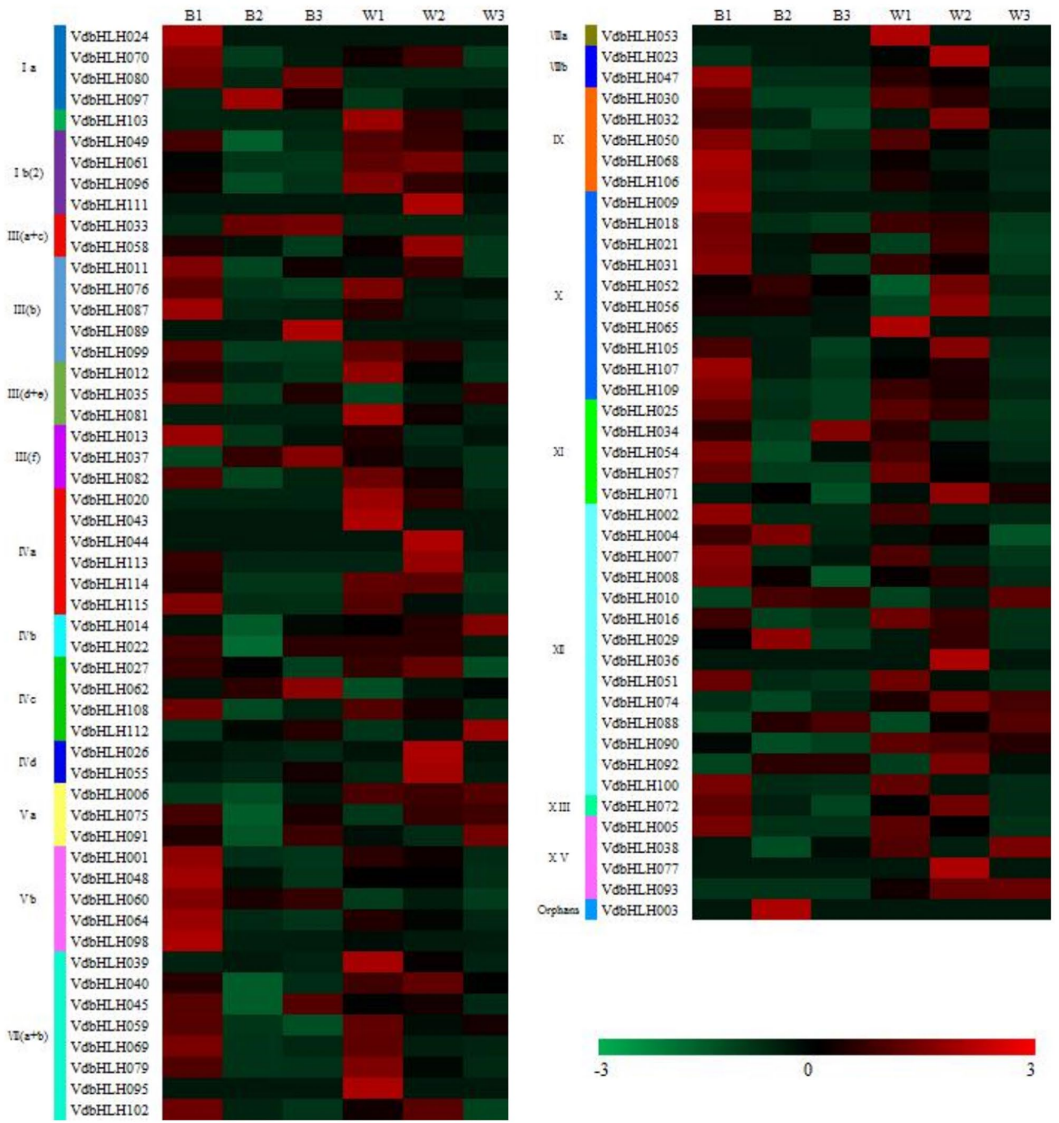
The heatmap of black and white spine grape berry *bHLH* genes at different developmental stages. *Note*: B1, B2 and B3 represent black spine grape berry of 40, 80 and 120 days after anthesis, respectively. W1, W2 and W3 represent white spine grape berry of 40, 80 and 120 days after anthesis, respectively.

We sequenced and analyzed the berry skins 40 (before veraison), 80 (at veraison) and 120 (berry ripening period) days after flowering of black and white spine grape. Among the 115 members of the *bHLH* family, 95 *bHLH* genes distributed in 21 subfamilies are expressed at least one of the developmental stages of the spine grape berry (Fig. 5). In order to screen the *bHLH* family candidate genes associated with anthocyanin synthesis and accumulation in pericarp of spine grape, reads per kilobase per million (RPKM) values were used to estimate the expression levels of *bHLH* family members. We screened 6 *bHLH* candidate genes (*VdbHLH003*, *VdbHLH004*, *VdbHLH033*, *VdbHLH037*, *VdbHLH062* and *VdbHLH097*) by differential gene expression pattern analysis. These genes were mainly expressed at 80 and 120 days after the flowering of the black spine grape, and the expression level was low or not expressed in the 40 days after the flowering of the black spine grape and in the white spine grape. We validated the candidate genes by qRT-PCR, which was consistent with the results of transcriptome sequencing (Supplementary Fig. S3). Among them, the expression levels of *VdbHLH033*, *VdbHLH037* and *VdbHLH062* increased with the ripening of blackberries. According to the previous analysis, *VdbHLH037* belongs to the subfamily III f. It is suggested that it may be related to anthocyanin synthesis in grapes.

### Interaction network prediction and functional analysis of candidate gene

**Figure 6:**
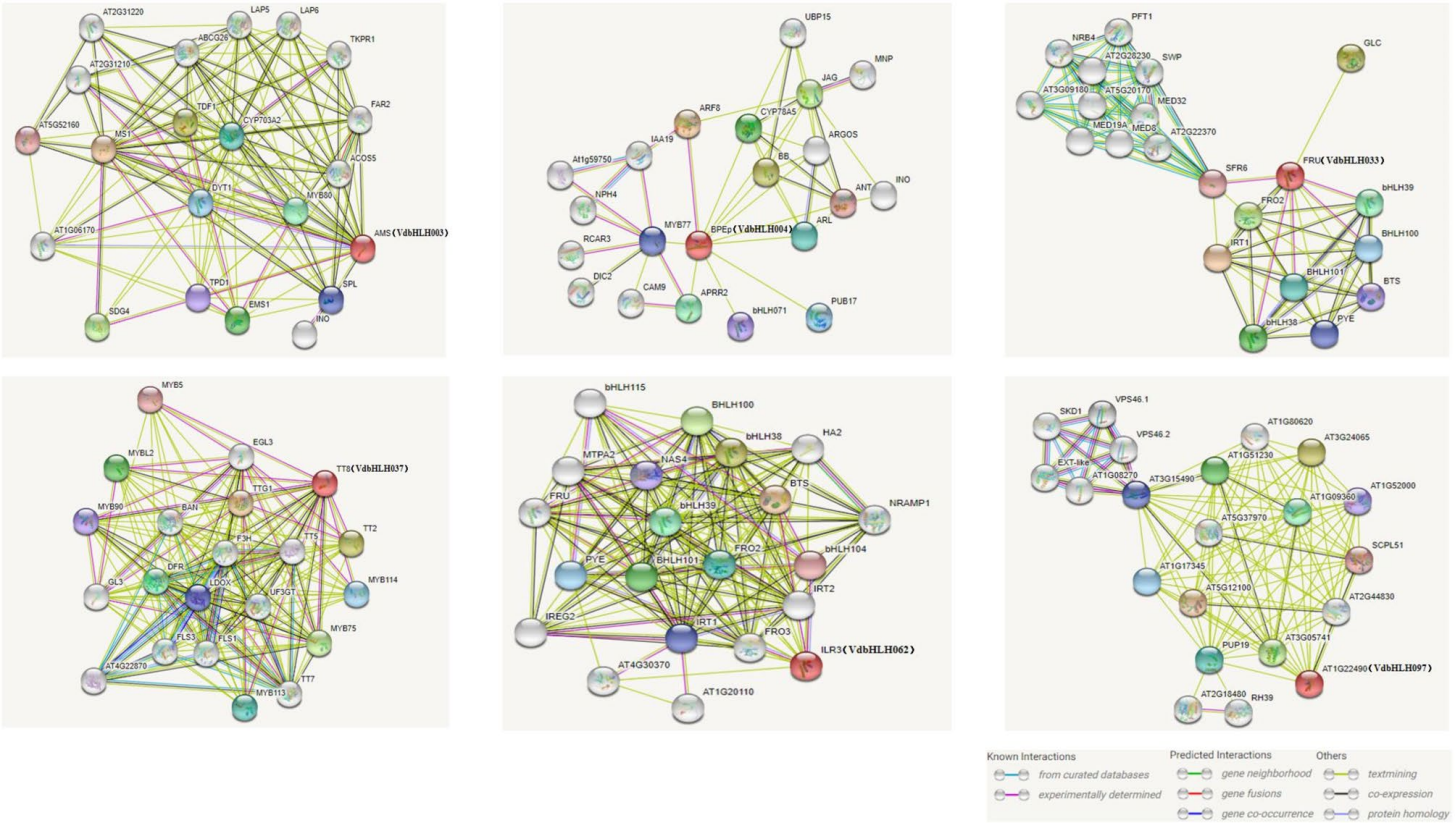
Interaction network analysis for *VdbHLH003*, *VdbHLH004*, *VdbHLH033*, *VdbHLH037*, *VdbHLH062* and *VdbHLH097*. *Note*: The predicted results are based on the orthologous gene in *Arabidopsis*. *VdbHLH* genes are shown in brackets.

Interaction network analysis helps us understand gene function and efficiency^21^. We used STRING to predict the interaction network of candidate genes based on *VdbHLH* orthologs in *Arabidopsis* (Fig. 6). *VdbHLH003* (*AMS* in *Arabidopsis*) plays a crucial role in tapetum development and is required for male fertility and pollen differentiation, especially during the post-meiotic transcriptional regulation of microspore development within the developing anther. The interaction between *VdbHLH004* (*BPEp* in *Arabidopsis*) and *ARF8* has been verified in *Arabidopsis*. Therefore, it is believed that *BPEp* influences cell size and ultimately controls petal size by regulating the expression of auxin response gene. *VdbHLH033* (*FRU* in *Arabidopsis*) is related to iron absorption. *VdbHLH037* (*TT8* in *Arabidopsis*) synergizes with *TT1*, *PAP1* and *TTG1* to regulate the flavonoid pathway. In addition, it also affects the expression of *DFR* (*dihydroflavonol 4-reductase*). The complex of *TT8*, *TT2* and *TTG1* is crucial to *BAN* genes that regulate flavonoid synthesis in seed endothelium. The genes *MYB113* and *MYB114* are involved in regulating anthocyanin synthesis. *F3H* (*Flavanone 3β-hydroxylase*), *LDOX* (*Leucoanthocyanidin dioxygenase*) and *UF3GT* (*UDP glucose-flavonoid 3-O-glucosyltransferase*) are enzymes that play a key role in the synthesis of anthocyanins. *VdbHLH062* (*ILR3* in *Arabidopsis*) plays a role in resistance to amide-linked indole-3-acetic acid (IAA) conjugates. The function of *VdbHLH097* (*AT1G22490* in *Arabidopsis*) is still unclear. These analyses suggest that *VdbHLH037* may be involved in anthocyanin synthesis.

### Overexpression of VdbHLH037 promoted anthocyanin accumulation of berry

**Figure 7:**
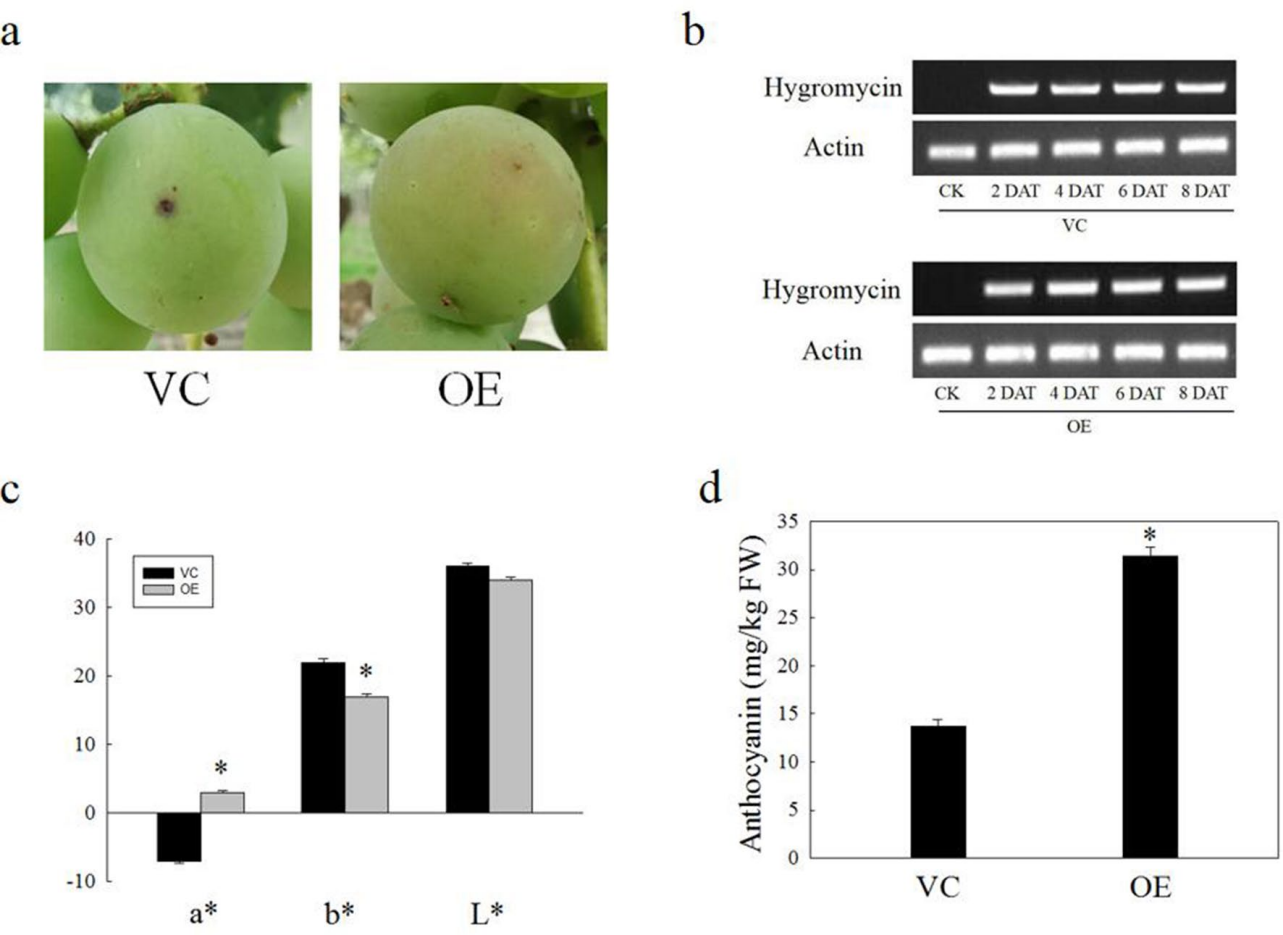
Transient expression result of ‘Kyoho’ berries. (**a**) ‘Kyoho’ fruits agroinfiltrated with VC (vector control) and OE (overexpression) at 6 days after injection. (**b**) Hygromycin gene (resistance marker gene) of pHB vector in fruits screened by RT-PCR. (**c**) Color parameters (L*, a*, b*) of VC and OE berries. (**d**) The total anthocyanin content of VC and OE berries. RT-PCR were normalized to the expression of *VlActin*.

Forty-five days after anthesis, green berry was used to inject *Agrobacterium* containing vector control (VC) and overexpression (OE) with a1-ml syringe. After eight days, the berries injected with OE showed faster color development than berries with VC (Fig. 7a). Compared with VC berries, OE berries had higher red color (a*), lower yellowness (b*) and similar lightness (L*) (Fig. 7c). The anthocyanin content of OE berries increased compared with VC berries (Fig. 7d). The number of red spots in OE berries was also more than that in VC berries after 8 days of infection (Supplementary Table S7). However, the number tended to be close after 16 days of injection (Supplementary Table S8). To further verify the results, RT-PCR was used to detect the infected berries. The results showed that the maker gene (*hygromycin* gene) could be detected 2, 4, 6, 8 days after infected by Agrobacterium containing OE and VC, but not by Agrobacterium alone (Fig. 7b). These results indicated that overexpression of *VdbHLH037* could promote anthocyanin accumulation in berries.

### Overexpression of VdbHLH037 can promote anthocyanin accumulation and upregulation of anthocyanin synthesis related genes in transgenic Arabidopsis

**Figure 8:**
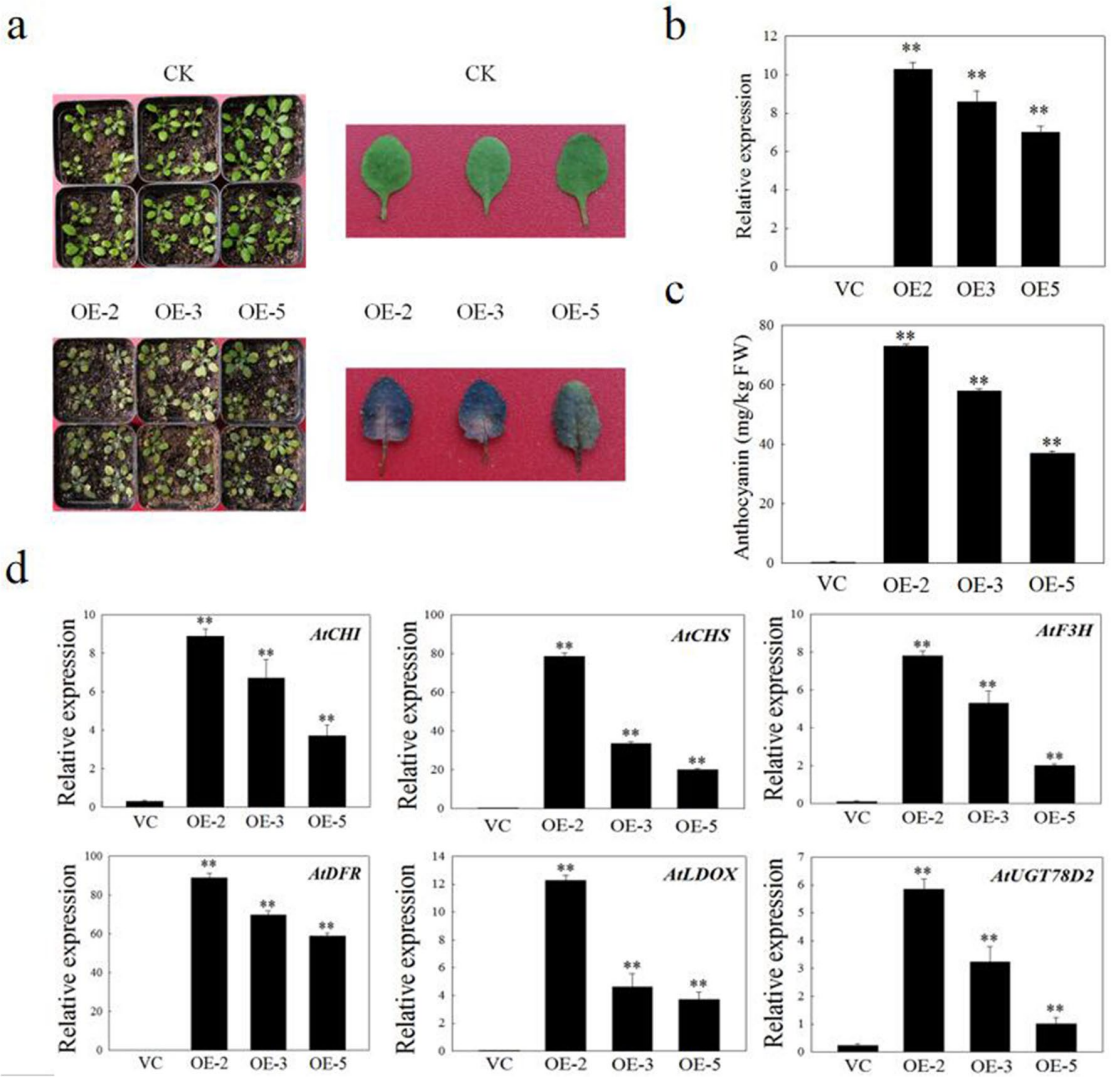
Functional analysis of *VdbHLH037* in transgenic *Arabidopsis*. (**a**) The phenotype of four-week-old transgenic *Arabidopsis* with VC and OE. (**b**) The transcription level of ***VdbHLH037*** in OE and VC transgenic Arabidopsis by qRT-PCR. (**c**) The total anthocyanin content of four-week-old transgenic *Arabidopsis* with VC and OE. (**d**) The transcription level of anthocyanin biosynthesis genes in transgenic *Arabidopsis* with VC and OE. qRT-PCR were normalized to the expression of *AtUBQ3*.

The T3-generation homozygous transgenic *Arabidopsis* was used to further analyze the function of *VdbHLH037* in anthocyanin synthesis. In four-week-old transgenic *Arabidopsis*, the leaves and petioles of the three OE lines (OE-2, OE-3 and OE-5) exhibited purple-black pigments when compared with VC lines (Fig. 8a). The anthocyanin content of three OE lines was also significantly higher than that of VC lines. There was no significant difference in rosette diameter and plant height between VC lines and OE lines (Fig. 8c). The transcription levels of 12 genes related to anthocyanin synthesis of VC and OE lines were analyzed. Compared with VC, the expression levels of *AtCHI*, *AtCHS*, *AtF3H*, *AtDFR*, *AtLDOX* (*leucoanthocyanidin dioxygenase*) and AtUGT78D2 in OE were significantly increased. The transcription level of OE-2 was the highest and that of OE-5 was the lowest. In VC, the expression level of these genes was low or barely detectable (Fig. 8d).

## Discussion

As one of the most important horticultural crops in the world, grape can be used as table grape or for wine, drying, juicing and so on. Grape color is the key factor for the commerciality of berry and also a key selection index for grape breeding. The color of berry is determined by the composition and content of anthocyanin. Previous studies have shown that *bHLH* is involved in anthocyanin synthesis, but the function of *bHLH* in grape coloration is rarely reported^23,24^. Spine grape has strong adaptability and resistance and is the only Chinese wild grape with both black and white berry, which provides an excellent material for studying the coloring mechanism of grape.

Based on the results of ‘Pinot Noir’ sequencing, We screened 137 *bHLH* genes, removed the pseudogenes through the analysis of the conserved domain and finally obtained 115 *bHLH* genes. We named them according to their positions on chromosomes (Fig. 1). Then we conducted a phylogenetic analysis of VdbHLH and divided it into 25 subfamilies, the results are similar to *Arabidopsis* and tomato^17,36^. According to the evolutionary relationship, *VdbHLH037* belongs to III (f) subfamily (Fig. 2). Members of this subfamily have been found to be related to anthocyanin synthesis. In three strawberry varieties, seven candidate *bHLH* genes related to anthocyanin synthesis were screened by genome-wide analysis of the bHLH family. In addition, it is considered that the *FabHLH29* gene of III (f) subfamily is related to anthocyanin synthesis through gene interaction network analysis and experimental verification^17^. In *Freesia hybrida*, *FhGL3L* and *FhTT8L* of III (f) subfamily are involved in flavonoid biosynthesis^48^. In chrysanthemums, *CmbHLH2* of III (f) subfamily regulates anthocyanin content by binding to the promoter of *CmDFR* and interacting with *CmMYB6* so that flowers appear red, pink and yellow^49^. In skins and seeds of grape, VvMYC1 regulates anthocyanin and proanthocyanidin synthesis through interacting with MYB5a, MYB5b, MYBA1/A2 and MYBPA1 to adjust genes of flavonoid pathway^50^. Through further analysis of gene structure and conserved motif, we added evidence supporting the phylogenetic relationship of the VdbHLH family (Supplementary Figs. S1 and S2). The basic region of the VdbHLH family determines the binding ability of DNA. The ratio of E-box-binding proteins in the VdbHLH family is lower than that in *Arabidopsis* and tomato, which indicates that binding motifs of the VdbHLH family are diverse (Fig. 3).

Through comparative genome analysis, we found that although some *bHLH* genes were lost during plant evolution and genome duplication events, the number of the *bHLH* gene was increasing (Fig. 4). In addition, it has been reported that there are more *bHLH* genes in higher plants than in lower plants^13^. These results indicated that *bHLH* genes play a key role in plant evolution. In order to further understand the function of *bHLH* genes in grape berry coloring, we analyzed the expression pattern of the bHLH family in the three periods of black and white spine grapes. The results showed that the expression level of *VdbHLH003*, *VdbHLH004*, *VdbHLH033*, *VdbHLH037*, *VdbHLH062* and *VdbHLH097* was significantly increased at the veraison stage of black spine grape, and was low or almost undetectable in the early development stage of black spine grape and the three development stages of white spine grape (Fig. 5). *VdbHLH037* is the highest up-regulated gene among the six genes, which was subsequently confirmed by qRT-PCR (Supplementary Fig. S3). Previously it was found that bHLH proteins perform their function by forming homodimer with bHLH protein or heterodimer with non- bHLH protein^51^. Therefore, we used *Arabidopsis* orthologs to predict the regulatory network of these six genes. The predicted interaction genes *F3H*, *DFR*, *LDOX* and *UF3GT* of the *VdbHLH037* gene were involved in the synthesis of anthocyanin. Besides, the interaction between MYB transcription factor and *VdbHLH037* is predicted (Fig. 6). Previous studies have shown that bHLH regulates anthocyanin biosynthesis by forming MBW transcriptional complex with MYB and WD40^52^. In addition, the sequence of *VdbHLH037* and AtTT8 is found to be highly similar. AtTT8 belongs to III (f) subfamily and regulates anthocyanin synthesis in *Arabidopsis* by forming MBW transcriptional complex with AtTT2 and AtTTG1^53^. The interaction network predicted by the remaining five genes had no direct relationship with the regulation process of anthocyanin synthesis. Accordingly, by the above results, it can be speculated that *VdbHLH037* plays a positive role at the veraison period in berry.

To understand the regulatory mechanism of *VdbHLH037* in anthocyanin synthesis of grape berry, we used the transient expression technology in berry to study its function^54^. After infection, compared with the VC, the coloration of OE berry was improved due to higher a* value. In addition, OE berries could accumulate more anthocyanin than VC. However, as time went on, the difference gradually decreased, which may be due to transient expression. Meanwhile, we also used transgenic *Arabidopsis* to study the function of *VdbHLH037* in the synthesis of anthocyanin. The results showed that OE-2, OE-3 and OE-5 could accumulate more anthocyanin than VC. The transcription level of *AtCHI*, *AtCHS*, *AtF3H*, *AtDFR*, *AtLDOX* and *AtUGT78D2* in OE berries was significantly higher than that in VC berries, and the expression level of these genes was consistent with the phenotype of transgenic *Arabidopsis*. AtCHI and AtCHS participated in the synthesis of flavonoid. AtF3H could catalyze and modify flavonoid. The flavonoid synthesis pathway entered the anthocyanin synthesis pathway under the action of *AtDFR* and *AtLDOX*. The gene *AtUGT78D2* was responsible for encoding the glycosyltransferase. These results suggested that *VdbHLH037* could promote anthocyanin accumulation by regulating related genes in the anthocyanin synthesis pathway. This study provides a theoretical basis for further understanding the function of bHLH family members in the process of grape berry coloring.

## Methods

### Identification and annotation of the grape bHLH family

The Hidden Markov Model (HMM) profile of the bHLH domain (PF00010) was downloaded from protein family (Pfam: http://pfam.sanger.ac.uk/). The PinotNoir PN40024 genome were downloaded from *EnsemblPlants* database (http://plants.ensembl.org/Multi/Tools/Blast?db=core) and were used as reference to blast the peptide with the BLASTp algorithm. All grape genes with non-redundant hits and expected values less than 0.001 were considered for further analysis. We obtained 137 *VdbHLH* genes. The 115 *VdbHLH* genes were screened by analyzing the conserved domain using conserved domains database (https://www.ncbi.nlm.nih.gov/Structure/cdd/wrpsb.cgi) and nucleotide and amino acid sequences using SMART (http://smart.embl-heidelberg.de/) and DNAStar. Details of 115 *VdbHLH* genes were obtained from the Plant Transcription Factor Database (http://planttfdb.cbi.pku.edu.cn/) and ExPASy Proteomics server (http://web.expasy.org/compute_pi/). The protein sequence of *Arabidopsis* and tomato was downloaded from Phytozome (https://phytozome.jgi.doe.gov/pz/portal.html).

### Bioinformatic analysis of bHLH family for grape

According to the distribution of the *bHLH* family on chromosome, we used MapDraw V2.1 to locate them on 19 chromosomes of grape. We analyzed the phylogenetic tree of the *bHLH* family by using iTOL (https://itol.embl.de/itol.cgi). The numbers are bootstrap values based on 1,000 iterations. Only bootstrap values larger than 50% support are displayed. We used GSDS2.0 (http://gsds.cbi.pku.edu.cn/) to analyze the gene structure and MEME (http://meme-suite.org/index.html) to analyze the conserved motif. Only motifs with an e-value < 1e^−20^ were retained for further analysis. We performed multiple sequence alignment of VdbHLH by CLUSTALW. The relationships of orthologous and paralogous *bHLH* genes among grape, *Arabidopsis* and tomato were constructed by OrthoMcl (https://orthomcl.org/orthomcl/). P-value cut-off of 1e^−5^ was chosen for all clusters constructed. The heatmap was drawn with Multiple experiment viewer (https://sourceforge.net/projects/mev-tm4/files/mev-tm4/). The interaction network of candidate genes was predicted by STRING (https://string-db.org/cgi/input.pl) with option value > 0.700.

### Transient expression of grape and transformation of Arabidopsis

The open reading frame (ORF) of *VdbHLH037* from spine grape was cloned into the BamHI site of the pHB vector with the Quick-Fusion Cloning Kit (biotool.cn) and a pair of specific primers (Supplementary Table S9). The recombinant plasmid and pHB vector were transformed into *Agrobacterium tumefaciens* strain GV3103. The ‘Kyoho’ were grown in China National Germplasm Resources Repository of Grape, Zhengzhou Fruit Research Institute, Chinese Academy of Agricultural Sciences. When the culture reached an OD600 of approximately 1.0, Agrobacterium cells were harvested and resuspended in infection buffer (10 mM MgCl2, 10 mM MES, pH 5.8, and 100 µM acetosyringone) and shaken for 4 h at 28 °C before being used for infiltration. Large green grape berries (45 DAA) were used and infected with a syringe until the whole fruit became hygrophanous. Approximately 2, 4, 6, and 8 days after treatment, the fruits infiltrated with the overexpression were harvested and used to detect the vector by RT-PCR^54^. For analysis of phenotypes, 200 fruits were injected, with 100 fruits being transformed with *VdbHLH037* and the other 100 used as the control.

The *VdbHLH037* ORF was cloned between NcoI and BglII sites of the pCAMBIA3301 vector with the Quick-Fusion Cloning Kit (biotool.cn) and a pair of specific primers (Supplementary Table S9). The recombinant plasmid and pCAMBIA3301 vector were transformed into *Aabidopsis* Columbia ecotype by the floral dip method^55^. The phosphinothricin resistance and qRT-PCR were used to screen seeds (Fig. 8B). Homozygous T3-generation plants were used for further analyses.

### Color measurement

The anthocyanin content in grape skins was measured after inoculation for 8 days. The leaf of four-week-old transgenic *Arabidopsis* with VC and OE were used to detect anthocyanin content. The samples (50 mg) was pulverized with liquid nitrogen and then each sample was homogenized in 1% (v/v) hydrochloric acid in methanol and shaken at 4 °C overnight. The anthocyanin content in grape and*Arabidopsis* were determined by measuring the absorbance of the extract at 530 nm using an ultraviolet–visible (UV–Vis) spectrophotometer^56^. The color of grape berry skin was measured after inoculation for 8 days with a handheld colorimeter (CR-400, Konica Minolta, Tokyo, Japan). Color parameters were recorded as L^∗^(lightness), a^∗^(redness) and b^∗^(yellowness).

### Analysis of qRT-PCR and RT-PCR

The **‘**Kyoho**’** skin of 2, 4, 6, 8 days after infected by Agrobacterium containing OE and VC were used to detect *hygromycin* gene. The berry skins at 40, 80 and 120 days after anthesis of black and white spine grape were used to identify candidate genes^57^. The RNA was extracted using TIANGEN RNAprep Pure Plant Kit (Tiangen Biotech). The four-week-old leaf of *Arabidopsis* was used to screen positive plants. The RNA was isolated from samples using TRIzol reagent (Invitrogen). Genomic DNA was removed by DNaseI (Thermo Scientific). The first-strand complementary DNA (cDNA) synthesis was performed with a RevertAid First-Strand cDNA Synthesis Kit (Thermo Scientific). qRT-PCR was performed in the presence of SYBR green qPCR Master Mix (Fermentas) and the amplification was performed in the Eco Real-Time PCR system (Illumina). All reactions were performed in triplicate^58^. The primers were designed using Oligo 7.0 and are listed in the Supplementary Table S9. The *VlActin* and *VdActin* were used as internal controls for grape. *AtUBQ3* was used as internal controls for *Arabidopsis*.

### Statistical analysis

All experiments were replicated independently at least three times, and the data were shown as mean ± SD of three independent experiments. Data were subjected to a statistical analysis according to Student’s t-test, and significant differences among the means were determined by the LSD (least significant difference), at*p < 0.05 and **p < 0.01.

## Supplementary Information

Supplementary Information
