# Supplementary material for: Genome-wide characterization and analysis of bHLH transcription factors related to anthocyanin biosynthesis in spine grapes (Vitis davidii)
Source: Sci Rep. 2021 Mar 25;11:6863. doi: 10.1038/s41598-021-85754-w (PMC7994560; doi:10.1038/s41598-021-85754-w)

**Cover page**

**Title:** Genome-wide characterization and analysis of bHLH transcription factors related to anthocyanin biosynthesis in spine grapes (*Vitis davidii*)

**Authors:** Ming Li^1^, Lei Sun^1^, Hong Gu^1^, Dawei Cheng^1^, XiZhi Guo^1^, Rui Chen^2^ , Zhiyong Wu^1^ , Jianfu Jiang^1^, Xiucai Fan^1^, Jinyong Chen^1^*

**Authors information:** Ming Li, E-mail: [liming07@caas.cn](mailto:Liming07@caas.cn); Lei Sun, E-mail: [slcaas@163.com](mailto:chenrui.taas@gmail.com); Hong Gu, E-mail: [guhong@caas.cn;](mailto:guhong@caas.cn;) Dawei Cheng, E-mail: [chengdawei@caas.cn](mailto:zhanghui06@caas.cn); XiZhi Guo, E-mail: [guoxizhi@caas.cn;](mailto:guoxizhi@caas.cn;) Rui Chen, E-mail: [chenrui.taas@gmail.com](mailto:chenrui.taas@gmail.com); Zhiyong Wu, E-mail: [13598072703@163.com;](mailto:13598072703@163.com;) Jianfu Jiang, E-mail: [jiangjianfu@caas.cn;](mailto:jiangjianfu@caas.cn;) Xiucai Fan,E-mail: fanxiucai@caas.cn; Jinyong Chen, E-mail: [chenjinyong@caas.cn](mailto:guoxizhi@caas.cn,).

**Affiliation:**

1. Zhengzhou Fruit Research Institute, Chinese Academy of Agricultural Sciences, Zhengzhou, 450009, China

2. Biotechnology Research Institute, Tianjin Academy of Agricultural Sciences, Tianjin, 300192, China

**First author:** Ming Li

E-mail: [liming07@caas.cn](mailto:Liming07@caas.cn)

*** Correspondence:** Jinyong Chen

E-mail: [chenjinyong@caas.cn,](mailto:chenjinyong@caas.cn,) Tel: +86-0371-65330948

**Fig S1. The structural features of *VdbHLH* transcription factors.**

The blue rectangles represent untranslated regions (UTRs). The yellow round-cornered rectangles represent exons. The black lines represent introns. The number above the lines represent the intron phase. Note: *VdbHLH006* is not listed because of its long sequence.


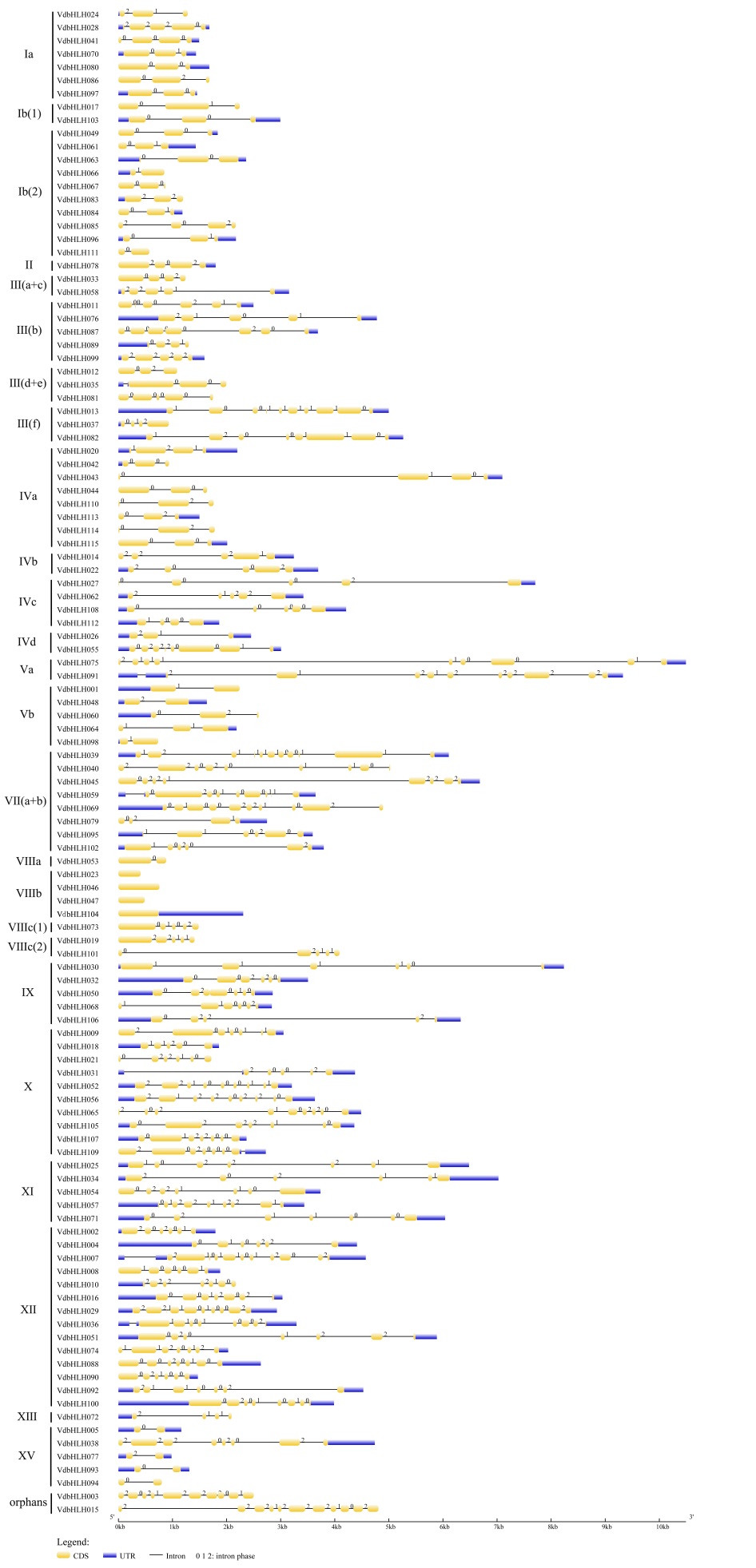


**Fig S2. The conservative motif analysis of *VdbHLH* family.**

The distribution of motifs in the *VdbHLH* family is represented by rectangles of different colors.


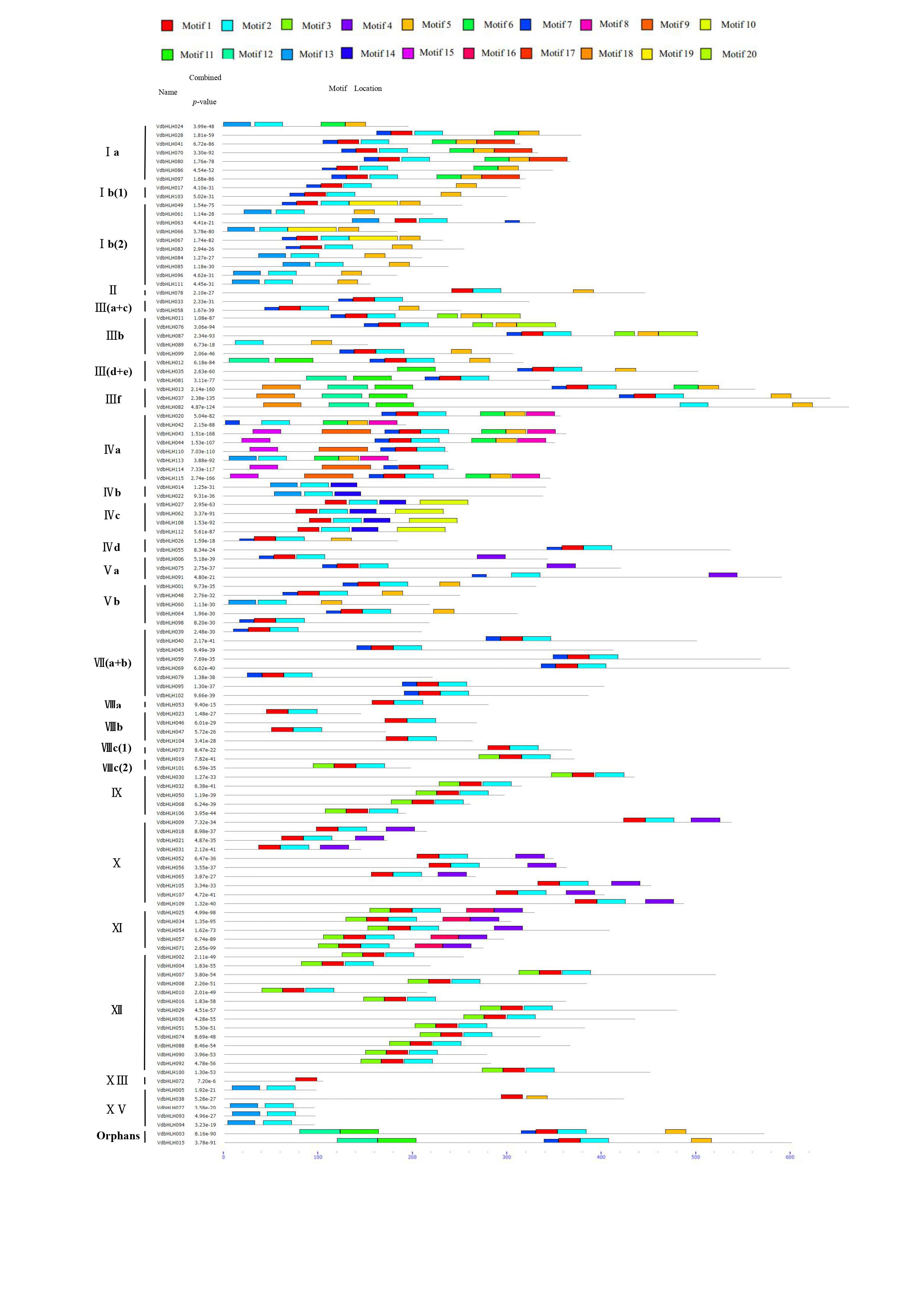


**Fig S3. Expression patterns of candidate genes in black and white spine grape berry at different developmental stages.**

Note: B1, B2 and B3 represent black spine grape berry of 40, 80 and 120 days after anthesis, respectively. W1, W2 and W3 represent white spine grape berry of 40, 80 and 120 days after anthesis, respectively. The experiment was independently repeated three times. Values represent the averages of three independent biological experiments, and error bars

represent standard deviations. qRT-PCR were normalized to the expression of *VdActin*.


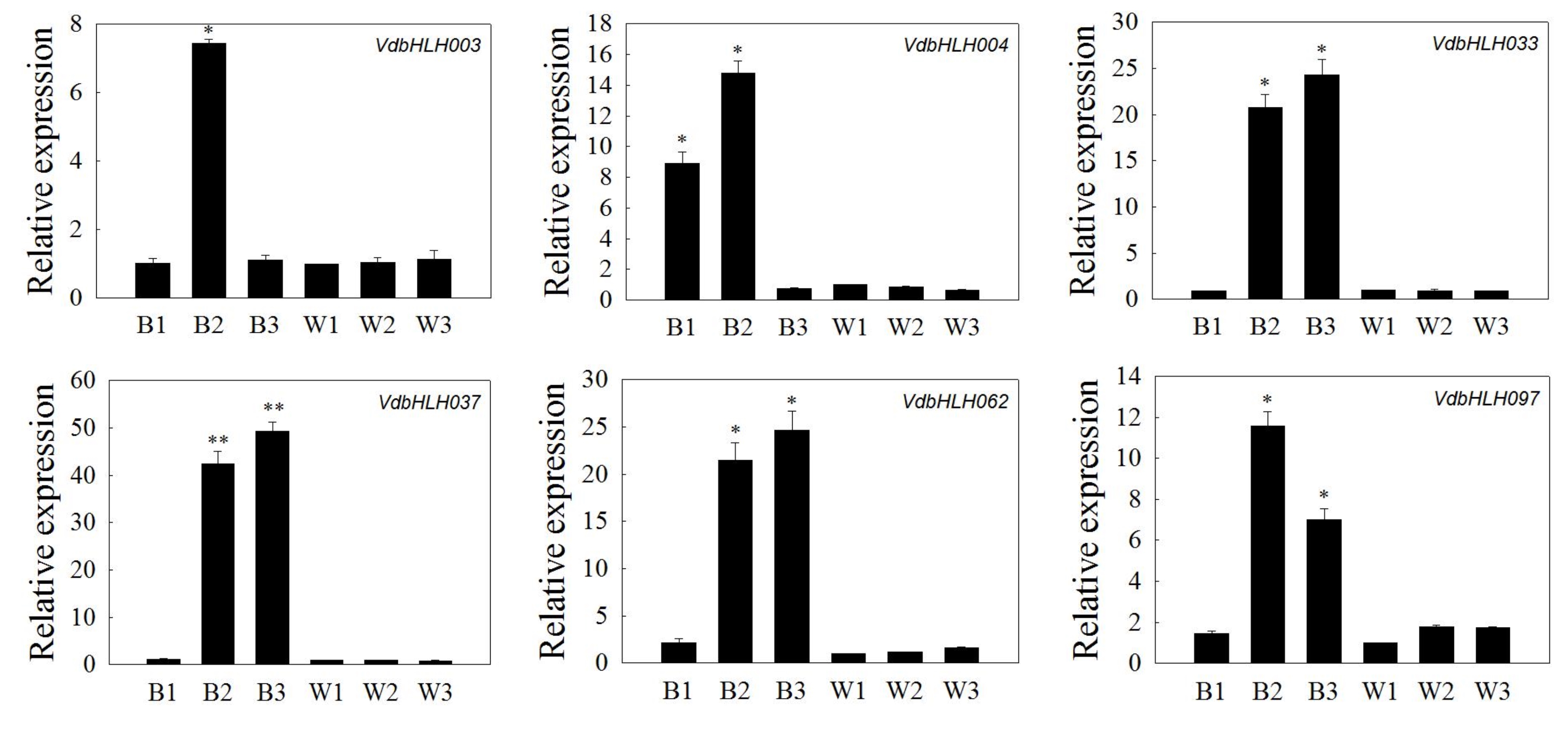


**Supplemental Table S1 Details of the *VdbHLH* family.**

**Gene locus is available in the National Center for Biotechnology Information Database. Mw, molecular weight; PI, isoelectric point.**

| Gene locus | **Gene symbol** | **ORF length(bp)** | **No. of aa** | **Mw(Da)** | **PI** | **Chr.** | **Location** | **Orientation** | **Group** |
| --- | --- | --- | --- | --- | --- | --- | --- | --- | --- |
| GSVIVT01011960001 | VdbHLH001 | 951 | 317 | 34659 | 6.9655 | 1 | 2606152..2608392 | forward | Ⅴb |
| GSVIVT01011873001 | VdbHLH002 | 726 | 242 | 26953.1 | 7.7879 | 1 | 3368552..3370256 | reverse | Ⅻ |
| GSVIVT01013720001 | VdbHLH003 | 1638 | 546 | 61083.1 | 4.8068 | 1 | 8215666..8218166 | reverse | Orphans |
| GSVIVT01020121001 | VdbHLH004 | 627 | 209 | 22804.3 | 8.707 | 1 | 10146181..10150591 | reverse | Ⅻ |
| GSVIVT01020027001 | VdbHLH005 | 279 | 93 | 10411.7 | 9.3627 | 1 | 11409315..11410477 | reverse | ⅩⅤ |
| GSVIVT01010100001 | VdbHLH006 | 987 | 329 | 36354.4 | 6.381 | 1 | 15386735..15409114 | reverse | Ⅴa |
| GSVIVT01010126001 | VdbHLH007 | 1491 | 497 | 53322.8 | 7.2526 | 1 | 15902530..15907102 | reverse | Ⅻ |
| GSVIVT01018942001 | VdbHLH008 | 1101 | 367 | 40643.6 | 5.8619 | 1 | 18512510..18514390 | forward | Ⅻ |
| GSVIVT01010280001 | VdbHLH009 | 1527 | 509 | 55767.9 | 6.37 | 1 | 18906768..18909820 | forward | Ⅹ |
| GSVIVT01004930001 | VdbHLH010 | 615 | 205 | 23040.2 | 8.0681 | 1 | 21582290..21584461 | reverse | Ⅻ |
| GSVIVT01004940001 | VdbHLH011 | 915 | 305 | 32939.7 | 10.3299 | 1 | 21696690..21699186 | forward | Ⅲ(b) |
| GSVIVT01019659001 | VdbHLH012 | 921 | 307 | 34366.4 | 7.5017 | 2 | 2286157..2287242 | forward | Ⅲ(d+e) |
| GSVIVT01019750001 | VdbHLH013 | 1632 | 544 | 61349.4 | 5.2486 | 2 | 2943365..2948362 | reverse | Ⅲ(f) |
| GSVIVT01024084001 | VdbHLH014 | 990 | 330 | 36266 | 6.2695 | 3 | 1252097..1255340 | reverse | Ⅳb |
| GSVIVT01024008001 | VdbHLH015 | 1722 | 574 | 64156.6 | 5.1867 | 3 | 1771005..1775813 | forward | Orphans |
| GSVIVT01023783001 | VdbHLH016 | 1038 | 346 | 37865.6 | 6.3719 | 3 | 3488694..3491726 | reverse | Ⅻ |
| GSVIVT01031718001 | VdbHLH017 | 918 | 306 | 32882.7 | 8.4553 | 3 | 3785857..3788100 | forward | Ⅰb(1) |
| GSVIVT01037903001 | VdbHLH018 | 609 | 203 | 21404.9 | 9.6689 | 3 | 6507405..6509263 | reverse | Ⅹ |
| GSVIVT01037846001 | VdbHLH019 | 1053 | 351 | 37968 | 4.3825 | 3 | 7192504..7193911 | forward | Ⅷc(2) |
| GSVIVT01036959001 | VdbHLH020 | 1035 | 345 | 38478.9 | 6.9267 | 3 | 9524910..9527107 | forward | Ⅳa |
| GSVIVT01018886001 | VdbHLH021 | 489 | 163 | 17555.2 | 10.5091 | 4 | 18962800..18964518 | reverse | Ⅹ |
| GSVIVT01018777001 | VdbHLH022 | 981 | 327 | 35994 | 5.323 | 4 | 20011688..20015380 | reverse | Ⅳb |
| GSVIVT01026516001 | VdbHLH023 | 411 | 137 | 15249.7 | 11.0449 | 4 | 22359047..22359457 | forward | Ⅷb |
| GSVIVT01017892001 | VdbHLH024 | 573 | 191 | 21332.7 | 8.6427 | 5 | 4441484..4442765 | reverse | Ⅰa |
| GSVIVT01018094001 | VdbHLH025 | 942 | 314 | 33323.6 | 5.8208 | 5 | 6435128..6441609 | forward | Ⅺ |
| GSVIVT01018111001 | VdbHLH026 | 531 | 177 | 20211.9 | 10.2717 | 5 | 6635387..6637839 | forward | Ⅳd |
| GSVIVT01027766001 | VdbHLH027 | 747 | 249 | 28193.8 | 6.5187 | 5 | 7500049..7507755 | reverse | Ⅳc |
| GSVIVT01021074001 | VdbHLH028 | 1104 | 368 | 41498.6 | 4.8409 | 5 | 14065432..14067113 | forward | Ⅰa |
| GSVIVT01021032001 | VdbHLH029 | 1374 | 458 | 49733.1 | 8.4582 | 5 | 15017218..15020147 | forward | Ⅻ |
| GSVIVT01013553001 | VdbHLH030 | 1233 | 411 | 44206.6 | 8.4063 | 5 | 20721906..20730141 | reverse | Ⅸ |
| GSVIVT01038142001 | VdbHLH031 | 411 | 137 | 15081.4 | 10.1311 | 5 | 23815690..23820063 | forward | Ⅹ |
| GSVIVT01025313001 | VdbHLH032 | 894 | 298 | 31759.4 | 9.4551 | 6 | 2129443..2132947 | reverse | Ⅸ |
| GSVIVT01031466001 | VdbHLH033 | 945 | 315 | 34918.6 | 4.9089 | 6 | 18252326..18253568 | forward | Ⅲ(a+c) |
| GSVIVT01000717001 | VdbHLH034 | 870 | 290 | 31102.3 | 6.3746 | 7 | 138160..145185 | forward | Ⅺ |
| GSVIVT01000771001 | VdbHLH035 | 1458 | 486 | 54700.6 | 6.9379 | 7 | 686589..688584 | forward | Ⅲ(d+e) |
| GSVIVT01007389001 | VdbHLH036 | 1248 | 416 | 45569.5 | 6.0934 | 7_random | 923263..926555 | forward | Ⅻ |
| GSVIVT01011123001 | VdbHLH037 | 1863 | 621 | 69303.6 | 5.7355 | 7 | 1386834..1392941 | reverse | Ⅲ(f) |
| GSVIVT01011109001 | VdbHLH038 | 1212 | 404 | 44851.7 | 6.5511 | 7 | 1499725..1504750 | forward | ⅩⅤ |
| GSVIVT01028238001 | VdbHLH039 | 603 | 201 | 20892.5 | 10.1203 | 7 | 4866937..4867868 | reverse | Ⅶ(a+b) |
| GSVIVT01028516001 | VdbHLH040 | 1440 | 480 | 52620.2 | 5.6748 | 7 | 9033294..9038033 | forward | Ⅶ(a+b) |
| GSVIVT01003437001 | VdbHLH041 | 918 | 306 | 34371.8 | 8.0206 | 7 | 15041110..15042602 | reverse | Ⅰa |
| GSVIVT01003701001 | VdbHLH042 | 561 | 187 | 21097.4 | 8.8674 | 7 | 15259649..15260585 | forward | Ⅳa |
| GSVIVT01003698001 | VdbHLH043 | 1053 | 351 | 39695.9 | 8.5556 | 7 | 15283280..15290377 | forward | Ⅳa |
| GSVIVT01003697001 | VdbHLH044 | 1017 | 339 | 37812.6 | 6.8848 | 7 | 15299383..15301022 | forward | Ⅳa |
| GSVIVT01022111001 | VdbHLH045 | 1185 | 395 | 43209.4 | 4.9456 | 7 | 16661373..16668053 | forward | Ⅶ(a+b) |
| GSVIVT01022120001 | VdbHLH046 | 759 | 253 | 27808.3 | 9.7761 | 7 | 16738987..16739745 | forward | Ⅷb |
| GSVIVT01030333001 | VdbHLH047 | 486 | 162 | 18161.4 | 6.9558 | 8 | 8753833..8754318 | reverse | Ⅷb |
| GSVIVT01025747001 | VdbHLH048 | 720 | 240 | 26492.9 | 6.6642 | 8 | 12276214..12277848 | forward | Ⅴb |
| GSVIVT01033618001 | VdbHLH049 | 738 | 246 | 27738.5 | 7.2161 | 8 | 19236350..19238185 | reverse | Ⅰb(2) |
| GSVIVT01033356001 | VdbHLH050 | 843 | 281 | 30748.4 | 9.7375 | 8 | 21244264..21247057 | reverse | Ⅸ |
| GSVIVT01033350001 | VdbHLH051 | 1065 | 365 | 40277.8 | 5.6402 | 8 | 21302624..21308510 | forward | Ⅻ |
| GSVIVT01017050001 | VdbHLH052 | 990 | 330 | 35750.8 | 6.7651 | 9 | 3786202..3789407 | forward | Ⅹ |
| GSVIVT01037927001 | VdbHLH053 | 795 | 265 | 30080.9 | 4.7584 | 10 | 11031132..11032016 | reverse | Ⅷa |
| GSVIVT01015006001 | VdbHLH054 | 1170 | 390 | 41353.4 | 6.6269 | 11 | 396544..400278 | reverse | Ⅺ |
| GSVIVT01015189001 | VdbHLH055 | 1542 | 514 | 57815.3 | 7.1472 | 11 | 1658052..1661058 | reverse | Ⅳd |
| GSVIVT01015353001 | VdbHLH056 | 1029 | 343 | 37487 | 6.8704 | 11 | 2919023..2922654 | forward | Ⅹ |
| GSVIVT01023585001 | VdbHLH057 | 849 | 283 | 29322.7 | 7.0435 | 11 | 7656325..7659760 | reverse | Ⅺ |
| GSVIVT01029311001 | VdbHLH058 | 735 | 245 | 27522.2 | 5.1169 | 11 | 17038389..17041543 | reverse | Ⅲ(a+c) |
| GSVIVT01020814001 | VdbHLH059 | 1635 | 545 | 59885 | 7.4542 | 12 | 1646903..1650547 | forward | Ⅶ(a+b) |
| GSVIVT01020688001 | VdbHLH060 | 627 | 209 | 22974.3 | 10.1835 | 12 | 3099863..3102455 | forward | Ⅴb |
| GSVIVT01020561001 | VdbHLH061 | 648 | 216 | 24156.1 | 5.3741 | 12 | 4344861..4346290 | reverse | Ⅰb(2) |
| GSVIVT01030640001 | VdbHLH062 | 672 | 224 | 24445.9 | 8.8581 | 12 | 7357660..7361079 | reverse | Ⅳc |
| GSVIVT01022881001 | VdbHLH063 | 963 | 321 | 36504.4 | 6.3752 | 12 | 18452410..18454770 | forward | Ⅰb(2) |
| GSVIVT01032789001 | VdbHLH064 | 894 | 298 | 33189.9 | 7.2658 | 13 | 764444..766628 | forward | Ⅴb |
| GSVIVT01016594001 | VdbHLH065 | 756 | 252 | 28909.1 | 9.4147 | 13 | 2545740..2550226 | reverse | Ⅹ |
| GSVIVT01034621001 | VdbHLH066 | 537 | 179 | 20332.6 | 10.1291 | 13 | 14129019..14129868 | reverse | Ⅰb(2) |
| GSVIVT01027323001 | VdbHLH067 | 678 | 226 | 25212.4 | 7.1644 | 13 | 16606926..16607794 | reverse | Ⅰb(2) |
| GSVIVT01032055001 | VdbHLH068 | 741 | 247 | 27599.3 | 9.9662 | 13 | 23164792..23167627 | forward | Ⅸ |
| GSVIVT01031338001 | VdbHLH069 | 1722 | 574 | 61533 | 6.8204 | 14 | 262785..267677 | reverse | Ⅶ(a+b) |
| GSVIVT01000012001 | VdbHLH070 | 972 | 324 | 36455.8 | 5.8111 | 14 | 2775388..2776822 | Reverse | Ⅰa |
| GSVIVT01021787001 | VdbHLH071 | 786 | 262 | 27990.9 | 7.7142 | 14 | 7430388..7436427 | forward | Ⅺ |
| GSVIVT01036202001 | VdbHLH072 | 600 | 100 | 11524.2 | 8.2012 | 14 | 9067763..9069855 | reverse | ⅩⅢ |
| GSVIVT01030846001 | VdbHLH073 | 1044 | 348 | 38463.9 | 6.3511 | 14 | 17518670..17520154 | forward | Ⅷc(1) |
| GSVIVT01031020001 | VdbHLH074 | 960 | 320 | 36016.8 | 8.3259 | 14 | 21028395..21030424 | forward | Ⅻ |
| GSVIVT01036533001 | VdbHLH075 | 1209 | 403 | 44354.2 | 5.9362 | 14 | 23125718..23136209 | forward | Ⅴa |
| GSVIVT01032998001 | VdbHLH076 | 1023 | 341 | 36661.8 | 9.0015 | 14 | 24969752..24974529 | reverse | Ⅲ(b) |
| GSVIVT01011380001 | VdbHLH077 | 273 | 91 | 10239.6 | 9.3627 | 14 | 29184240..29185221 | reverse | ⅩⅤ |
| GSVIVT01011330001 | VdbHLH078 | 1302 | 434 | 48789.4 | 5.6095 | 15 | 8616820..8618617 | reverse | Ⅱ |
| GSVIVT01018165001 | VdbHLH079 | 636 | 212 | 23433.4 | 10.8278 | 15 | 13708127..13710873 | reverse | Ⅶ(a+b) |
| GSVIVT01027446001 | VdbHLH080 | 1071 | 357 | 39767.5 | 7.3996 | 15 | 16674193..16675872 | reverse | Ⅰa |
| GSVIVT01027412001 | VdbHLH081 | 1002 | 334 | 36695.4 | 5.4313 | 15 | 16926728..16928476 | reverse | Ⅲ(d+e) |
| GSVIVT01026927001 | VdbHLH082 | 1920 | 640 | 71999.3 | 4.9851 | 15 | 19338784..19344048 | reverse | Ⅲ(f) |
| GSVIVT01018461001 | VdbHLH083 | 744 | 248 | 27741.8 | 9.5874 | 16 | 14744889..14746085 | forward | Ⅰb(2) |
| GSVIVT01018460001 | VdbHLH084 | 615 | 205 | 22685 | 7.7559 | 16 | 14755512..14756695 | forward | Ⅰb(2) |
| GSVIVT01018459001 | VdbHLH085 | 696 | 232 | 25944.7 | 10.0098 | 16 | 14775868..14778037 | forward | Ⅰb(2) |
| GSVIVT01028719001 | VdbHLH086 | 1017 | 339 | 36986.8 | 5.1653 | 16 | 19472593..19474275 | forward | Ⅰa |
| GSVIVT01008637001 | VdbHLH087 | 1458 | 486 | 52796.2 | 5.5362 | 17 | 232691..236378 | reverse | Ⅲ(b) |
| GSVIVT01008628001 | VdbHLH088 | 1050 | 350 | 38748.6 | 6.874 | 17 | 299665..302297 | forward | Ⅻ |
| GSVIVT01008305001 | VdbHLH089 | 444 | 148 | 16557.1 | 6.5279 | 17 | 3428366..3429668 | reverse | Ⅲ(b) |
| GSVIVT01008299001 | VdbHLH090 | 798 | 266 | 29353.7 | 6.1467 | 17 | 3477176..3478644 | forward | Ⅻ |
| GSVIVT01008164001 | VdbHLH091 | 1698 | 566 | 61847.6 | 8.0563 | 17 | 5177472..5186799 | forward | Ⅴa |
| GSVIVT01008093001 | VdbHLH092 | 810 | 270 | 29063.2 | 5.468 | 17 | 5834789..5839316 | reverse | Ⅻ |
| GSVIVT01008034001 | VdbHLH093 | 276 | 92 | 10198.4 | 9.3634 | 17 | 6455525..6456835 | reverse | ⅩⅤ |
| GSVIVT01008030001 | VdbHLH094 | 273 | 91 | 10328.7 | 9.3646 | 17 | 6493136..6493937 | forward | ⅩⅤ |
| GSVIVT01007914001 | VdbHLH095 | 1158 | 386 | 42207.8 | 9.2609 | 17 | 7609546..7613137 | forward | Ⅶ(a+b) |
| GSVIVT01007772001 | VdbHLH096 | 537 | 179 | 20178.5 | 9.9624 | 17 | 9199303..9201475 | reverse | Ⅰb(2) |
| GSVIVT01009097001 | VdbHLH097 | 933 | 311 | 34622 | 6.2593 | 18 | 5009692..5011145 | reverse | Ⅰa |
| GSVIVT01009166001 | VdbHLH098 | 627 | 209 | 22405.1 | 9.7096 | 18 | 5668081..5668815 | reverse | Ⅴb |
| GSVIVT01009234001 | VdbHLH099 | 888 | 296 | 33815.4 | 4.7389 | 18 | 6502785..6504375 | forward | Ⅲ(b) |
| GSVIVT01009292001 | VdbHLH100 | 1293 | 431 | 46341.6 | 6.3546 | 18 | 7033471..7037456 | reverse | Ⅻ |
| GSVIVT01009357001 | VdbHLH101 | 561 | 187 | 20751.4 | 9.8804 | 18 | 7711468..7715556 | reverse | Ⅷc(2) |
| GSVIVT01009467001 | VdbHLH102 | 1110 | 370 | 39666 | 6.5099 | 18 | 8609031..8612826 | forward | Ⅶ(a+b) |
| GSVIVT01009469001 | VdbHLH103 | 876 | 292 | 31906.9 | 7.9017 | 18 | 8624953..8627945 | forward | Ⅰb(1) |
| GSVIVT01009479001 | VdbHLH104 | 747 | 249 | 27435.5 | 9.419 | 18 | 8724790..8727096 | forward | Ⅷb |
| GSVIVT01009761001 | VdbHLH105 | 1284 | 428 | 46032.6 | 8.0373 | 18 | 11056811..11061172 | reverse | Ⅹ |
| GSVIVT01026056001 | VdbHLH106 | 546 | 182 | 20155.7 | 8.9523 | 18 | 25210556..25216882 | forward | Ⅸ |
| GSVIVT01014542001 | VdbHLH107 | 1155 | 385 | 41725.1 | 7.197 | 19 | 4971654..4974022 | reverse | Ⅹ |
| GSVIVT01014589001 | VdbHLH108 | 714 | 238 | 25960.4 | 8.1891 | 19 | 5400650..5404859 | forward | Ⅳc |
| GSVIVT01005301001 | VdbHLH109 | 1395 | 465 | 50943.8 | 5.7572 | un | 20415278..20418002 | forward | Ⅹ |
| GSVIVT01006588001 | VdbHLH110 | 690 | 230 | 25717.6 | 10.157 | un | 26860663..26862424 | forward | Ⅳa |
| GSVIVT01007117001 | VdbHLH111 | 456 | 152 | 17333.6 | 6.8075 | un | 30153816..30154387 | forward | Ⅰb(2) |
| GSVIVT01007339001 | VdbHLH112 | 678 | 226 | 25246.4 | 9.8418 | un | 31435805..31437668 | forward | Ⅳc |
| GSVIVT01002668001 | VdbHLH113 | 534 | 178 | 20204.4 | 9.9536 | un | 35805535..35807033 | reverse | Ⅳa |
| GSVIVT01002833001 | VdbHLH114 | 708 | 236 | 26371.2 | 9.7736 | un | 36876661..36878443 | reverse | Ⅳa |
| GSVIVT01004409001 | VdbHLH115 | 1005 | 335 | 37421.1 | 8.3656 | un | 38434334..38436345 | reverse | Ⅳa |

**Supplemental Table S2 Normal expression sequences of 20 motifs identified.**

Sites represent the time of motif appears.

| **Sites** | **Length(aa)** |
| --- | --- |
| 96 | 22 |
| 113 | 29 |
| 29 | 21 |
| 18 | 29 |
| 44 | 21 |
| 14 | 25 |
| 49 | 15 |
| 6 | 29 |
| 4 | 50 |
| 4 | 49 |
| 8 | 39 |
| 7 | 41 |
| 16 | 28 |
| 6 | 27 |
| 5 | 29 |
| 4 | 28 |
| 4 | 39 |
| 3 | 39 |
| 3 | 50 |
| 3 | 40 |

**Supplemental Table S3 Conserved amino acid frequency of the bHLH domain.**

Percentage of conserved amino acids in 115 VdbHLH domains based on previous results.

|  | Atchley et al. | | Toledo-Ortiz et al. | | This study | |
| --- | --- | --- | --- | --- | --- | --- |
|  | **Position in the alignment** | **Consensus motif amino acid frequency within the bHLH domain** | **Position in the alignment** | **Amino acid frequency within the *Arabidopsis bHLH domain*** | **Position in the alignment** | **Amino acid frequency within the *Vitis davidii bHLH domain*** |
| Basic | 1 | R (61%), K (27%) | 1 | R (24%), K (22%) | 1 | S (5%), K (5%) |
|  | 2 | R (77%), K (16%) | 2 | R (35%) | 2 | R (7%) |
|  | 9 | E (93%) | 13 | E (76%), A (10%) | 13 | E (77%) |
|  | 10 | R (81%), K (14%) | 14 | R (74%), K (14%) | 14 | R (73%), K (20%) |
|  | 12 | R (91%) | 16 | R (91%) | 16 | R (92%) |
| Helix | 16 | I (35%), L (33%), V (23%) | 20 | I (52%), L (27%), M (12%) | 20 | I (50%), L (30%), M (15%) |
|  | 17 | N (74%) | 21 | N (51%), S (19%) | 21 | N (48%), S (21%) |
|  | 20 | F (72%), L (14%), I (9%) | 24 | F (26%), L (26%), M (20%), I (14%) | 26 | L (24%), F (23%), M (23%), I (14%) |
|  | 23 | L (98%) | 27 | L (100%) | 29 | L (97%) |
|  | 24 | R (44%), K (35%) | 28 | Q (42%), R (35%) | 30 | Q (44%), R (35%) |
| Loop | 47 | K (58%), R (24%) | 39 | K (66%) | 38 | K (43%) |
| Helix | 50 | K (93%) | 42 | K (45%), T (13%) | 51 | K (45%), T (17%) |
|  | 53 | I (74%), T (15%), V (7%) | 45 | M (33%), I (27%), V (16%), L(14%) | 54 | M (30%), I (25%), V (25%), L(7%) |
|  | 54 | L (98%) | 46 | L (76%), V(14%) | 55 | L (80%), V(7%) |
|  | 57 | A (76%) | 49 | A (60%), I (16%), V (12%) | 58 | A (57%), I (17%), V (14%) |
|  | 58 | I (31%), V (27%), T (23%) | 50 | I (63%), V (22%) | 59 | I (61%), V (23%) |
|  | 60 | Y (77%) | 52 | Y (78%) | 61 | Y (80%) |
|  | 61 | I (69%), L (16%), V (8%) | 53 | I (40%), V (33%), L (13%) | 62 | I (37%), V (37%), L (23%) |
|  | 64 | L (80%), M (7%) | 56 | L (93%) | 65 | L (98%) |

**Supplemental Table S4 Prediction of DNA binding type based on conserved amino acid residues in the basic region.**

| **Gene symbol** | **9** | **13** | **16** | **17** | **Type** |
| --- | --- | --- | --- | --- | --- |
| VdbHLH001 | H | E | R | R | G-box |
| VdbHLH002 | H | E | R | R | G-box |
| VdbHLH003 | N | E | R | R | Non-G-box |
| VdbHLH004 | H | E | R | R | G-box |
| VdbHLH005 | P | S | S | D | Non-E-box |
| VdbHLH006 | H | E | R | R | G-box |
| VdbHLH007 | H | E | R | R | G-box |
| VdbHLH008 | H | E | R | R | G-box |
| VdbHLH009 | S | M | P | K | Non-E-box |
| VdbHLH010 | H | E | R | R | G-box |
| VdbHLH011 | N | E | R | R | Non-G-box |
| VdbHLH012 | H | E | R | R | G-box |
| VdbHLH013 | H | E | R | R | G-box |
| VdbHLH014 | K | E | R | R | G-box |
| VdbHLH015 | N | E | R | R | Non-G-box |
| VdbHLH016 | H | E | R | R | G-box |
| VdbHLH017 | H | E | R | R | G-box |
| VdbHLH018 | S | V | K | K | Non-E-box |
| VdbHLH019 | Q | A | R | R | Non-E-box |
| VdbHLH020 | H | E | R | R | G-box |
| VdbHLH021 | S | L | R | K | Non-E-box |
| VdbHLH022 | K | E | R | R | G-box |
| VdbHLH023 | Q | A | R | R | Non-E-box |
| VdbHLH024 | H | E | R | R | G-box |
| VdbHLH025 | H | E | R | R | G-box |
| VdbHLH026 | H | E | R | R | G-box |
| VdbHLH027 | K | E | R | R | G-box |
| VdbHLH028 | H | E | R | R | G-box |
| VdbHLH029 | H | E | R | R | G-box |
| VdbHLH030 | R | E | R | R | Non-G-box |
| VdbHLH031 | I | T | R | K | Non-E-box |
| VdbHLH032 | R | E | R | R | Non-G-box |
| VdbHLH033 | T | E | R | R | Non-G-box |
| VdbHLH034 | H | E | R | R | G-box |
| VdbHLH035 | H | E | R | R | G-box |
| VdbHLH036 | H | E | R | R | G-box |
| VdbHLH037 | H | E | R | R | G-box |
| VdbHLH038 | A | D | R | R | Non-E-box |
| VdbHLH039 | H | E | R | R | G-box |
| VdbHLH040 | H | E | R | R | G-box |
| VdbHLH041 | H | E | R | R | G-box |
| VdbHLH042 | H | E | R | R | G-box |
| VdbHLH043 | H | E | R | R | G-box |
| VdbHLH044 | H | E | R | R | G-box |
| VdbHLH045 | H | E | R | R | G-box |
| VdbHLH046 | Q | A | R | R | Non-E-box |
| VdbHLH047 | E | M | P | Y | Non-E-box |
| VdbHLH048 | H | E | R | R | G-box |
| VdbHLH049 | H | E | R | R | G-box |
| VdbHLH050 | R | E | R | R | Non-G-box |
| VdbHLH051 | H | E | R | R | G-box |
| VdbHLH052 | - | L | R | K | Non-E-box |
| VdbHLH053 | Q | A | R | R | Non-E-box |
| VdbHLH054 | H | E | R | R | G-box |
| VdbHLH055 | H | E | R | R | G-box |
| VdbHLH056 | - | F | R | K | Non-E-box |
| VdbHLH057 | H | E | R | R | G-box |
| VdbHLH058 | N | E | R | R | Non-G-box |
| VdbHLH059 | H | E | R | R | G-box |
| VdbHLH060 | H | E | R | R | G-box |
| VdbHLH061 | R | E | R | R | Non-G-box |
| VdbHLH062 | K | E | R | R | Non-G-box |
| VdbHLH063 | H | E | R | R | G-box |
| VdbHLH064 | H | E | R | R | G-box |
| VdbHLH065 | R | P | R | S | Non-E-box |
| VdbHLH066 | H | V | R | R | Non-E-box |
| VdbHLH067 | H | E | R | R | G-box |
| VdbHLH068 | R | E | R | R | Non-G-box |
| VdbHLH069 | H | E | R | R | G-box |
| VdbHLH070 | H | E | R | R | G-box |
| VdbHLH071 | H | E | R | R | G-box |
| VdbHLH072 | S | N | V | V | Non-E-box |
| VdbHLH073 | Q | A | R | R | Non-E-box |
| VdbHLH074 | H | E | R | R | G-box |
| VdbHLH075 | H | E | R | R | G-box |
| VdbHLH076 | N | E | R | R | Non-G-box |
| VdbHLH077 | S | S | S | D | Non-E-box |
| VdbHLH078 | S | E | R | R | Non-G-box |
| VdbHLH079 | H | E | R | R | G-box |
| VdbHLH080 | H | E | R | R | G-box |
| VdbHLH081 | H | E | R | R | G-box |
| VdbHLH082 | H | D | R | - | Non-E-box |
| VdbHLH083 | H | E | R | R | G-box |
| VdbHLH084 | R | E | R | R | Non-G-box |
| VdbHLH085 | R | E | R | R | Non-G-box |
| VdbHLH086 | H | E | R | R | G-box |
| VdbHLH087 | N | E | R | R | Non-G-box |
| VdbHLH088 | H | E | R | R | G-box |
| VdbHLH089 | - | - | - | - | Non-DNA-binding |
| VdbHLH090 | H | E | R | R | G-box |
| VdbHLH091 | H | E | R | R | G-box |
| VdbHLH092 | H | E | R | R | G-box |
| VdbHLH093 | S | S | T | D | Non-E-box |
| VdbHLH094 | - | S | T | D | Non-E-box |
| VdbHLH095 | H | E | R | R | G-box |
| VdbHLH096 | R | E | R | R | Non-G-box |
| VdbHLH097 | H | E | R | R | G-box |
| VdbHLH098 | H | E | R | R | G-box |
| VdbHLH099 | N | E | R | R | Non-G-box |
| VdbHLH100 | H | E | R | R | G-box |
| VdbHLH101 | Q | A | R | R | Non-E-box |
| VdbHLH102 | H | E | R | R | G-box |
| VdbHLH103 | H | E | R | R | G-box |
| VdbHLH104 | Q | A | R | R | Non-E-box |
| VdbHLH105 | S | I | R | K | Non-E-box |
| VdbHLH106 | R | E | R | R | Non-E-box |
| VdbHLH107 | P | F | R | K | Non-E-box |
| VdbHLH108 | K | E | R | R | G-box |
| VdbHLH109 | P | F | R | K | Non-E-box |
| VdbHLH110 | H | E | R | R | G-box |
| VdbHLH111 | R | E | R | R | Non-G-box |
| VdbHLH112 | K | E | R | R | G-box |
| VdbHLH113 | H | E | R | R | G-box |
| VdbHLH114 | H | E | R | R | G-box |
| VdbHLH115 | H | E | R | R | G-box |

**Supplemental Table S5 The orthologous *bHLH* genes in grape, *Arabidopsis* and tomato genome.**

| **bHLH Name1** | **bHLH Name2** |
| --- | --- |
| VdbHLH001 | AtbHLH079 |
| VdbHLH003 | AtbHLH041 |
| VdbHLH004 | AtbHLH146 |
| VdbHLH005 | AtbHLH039 |
| VdbHLH007 | AtbHLH027 |
| VdbHLH008 | AtbHLH016 |
| VdbHLH012 | AtbHLH022 |
| VdbHLH012 | AtbHLH134 |
| VdbHLH012 | AtbHLH135 |
| VdbHLH013 | AtbHLH030 |
| VdbHLH014 | AtbHLH117 |
| VdbHLH015 | AtbHLH041 |
| VdbHLH019 | AtbHLH018 |
| VdbHLH028 | AtbHLH078 |
| VdbHLH029 | AtbHLH016 |
| VdbHLH032 | AtbHLH026 |
| VdbHLH035 | AtbHLH101 |
| VdbHLH040 | AtbHLH008 |
| VdbHLH041 | AtbHLH038 |
| VdbHLH041 | AtbHLH133 |
| VdbHLH042 | AtbHLH046 |
| VdbHLH043 | AtbHLH046 |
| VdbHLH044 | AtbHLH046 |
| VdbHLH046 | AtbHLH083 |
| VdbHLH049 | AtbHLH059 |
| VdbHLH050 | AtbHLH026 |
| VdbHLH051 | AtbHLH087 |
| VdbHLH052 | AtbHLH017 |
| VdbHLH053 | AtbHLH020 |
| VdbHLH056 | AtbHLH112 |
| VdbHLH057 | AtbHLH048 |
| VdbHLH059 | AtbHLH062 |
| VdbHLH061 | AtbHLH103 |
| VdbHLH062 | AtbHLH025 |
| VdbHLH062 | AtbHLH076 |
| VdbHLH062 | AtbHLH141 |
| VdbHLH067 | AtbHLH059 |
| VdbHLH069 | AtbHLH119 |
| VdbHLH070 | AtbHLH038 |
| VdbHLH070 | AtbHLH133 |
| VdbHLH077 | AtbHLH039 |
| VdbHLH078 | AtbHLH006 |
| VdbHLH078 | AtbHLH053 |
| VdbHLH081 | AtbHLH022 |
| VdbHLH081 | AtbHLH134 |
| VdbHLH081 | AtbHLH135 |
| VdbHLH082 | AtbHLH030 |
| VdbHLH084 | AtbHLH029 |
| VdbHLH084 | AtbHLH106 |
| VdbHLH085 | AtbHLH029 |
| VdbHLH085 | AtbHLH106 |
| VdbHLH086 | AtbHLH140 |
| VdbHLH087 | AtbHLH080 |
| VdbHLH089 | AtbHLH125 |
| VdbHLH089 | AtbHLH148 |
| VdbHLH092 | AtbHLH146 |
| VdbHLH093 | AtbHLH039 |
| VdbHLH095 | AtbHLH008 |
| VdbHLH097 | AtbHLH038 |
| VdbHLH097 | AtbHLH133 |
| VdbHLH100 | AtbHLH137 |
| VdbHLH101 | AtbHLH018 |
| VdbHLH102 | AtbHLH008 |
| VdbHLH103 | AtbHLH024 |
| VdbHLH104 | AtbHLH083 |
| VdbHLH107 | AtbHLH028 |
| VdbHLH108 | AtbHLH025 |
| VdbHLH108 | AtbHLH076 |
| VdbHLH108 | AtbHLH141 |
| VdbHLH001 | SlbHLH058 |
| VdbHLH002 | SlbHLH046 |
| VdbHLH002 | SlbHLH094 |
| VdbHLH002 | SlbHLH153 |
| VdbHLH003 | SlbHLH110 |
| VdbHLH006 | SlbHLH048 |
| VdbHLH006 | SlbHLH091 |
| VdbHLH007 | SlbHLH001 |
| VdbHLH007 | SlbHLH056 |
| VdbHLH007 | SlbHLH075 |
| VdbHLH008 | SlbHLH046 |
| VdbHLH008 | SlbHLH094 |
| VdbHLH008 | SlbHLH153 |
| VdbHLH011 | SlbHLH090 |
| VdbHLH011 | SlbHLH155 |
| VdbHLH013 | SlbHLH115 |
| VdbHLH013 | SlbHLH124 |
| VdbHLH015 | SlbHLH110 |
| VdbHLH016 | SlbHLH021 |
| VdbHLH019 | SlbHLH140 |
| VdbHLH021 | SlbHLH069 |
| VdbHLH022 | SlbHLH022 |
| VdbHLH023 | SlbHLH032 |
| VdbHLH023 | SlbHLH117 |
| VdbHLH028 | SlbHLH006 |
| VdbHLH028 | SlbHLH148 |
| VdbHLH029 | SlbHLH046 |
| VdbHLH029 | SlbHLH094 |
| VdbHLH029 | SlbHLH153 |
| VdbHLH031 | SlbHLH089 |
| VdbHLH031 | SlbHLH109 |
| VdbHLH032 | SlbHLH131 |
| VdbHLH032 | SlbHLH149 |
| VdbHLH032 | SlbHLH158 |
| VdbHLH034 | SlbHLH125 |
| VdbHLH035 | SlbHLH096 |
| VdbHLH037 | SlbHLH115 |
| VdbHLH037 | SlbHLH124 |
| VdbHLH040 | SlbHLH038 |
| VdbHLH040 | SlbHLH049 |
| VdbHLH040 | SlbHLH067 |
| VdbHLH040 | SlbHLH097 |
| VdbHLH040 | SlbHLH122 |
| VdbHLH040 | SlbHLH130 |
| VdbHLH040 | SlbHLH157 |
| VdbHLH041 | SlbHLH006 |
| VdbHLH041 | SlbHLH148 |
| VdbHLH042 | SlbHLH037 |
| VdbHLH043 | SlbHLH037 |
| VdbHLH044 | SlbHLH037 |
| VdbHLH046 | SlbHLH032 |
| VdbHLH046 | SlbHLH117 |
| VdbHLH047 | SlbHLH032 |
| VdbHLH047 | SlbHLH117 |
| VdbHLH049 | SlbHLH144 |
| VdbHLH049 | SlbHLH145 |
| VdbHLH050 | SlbHLH131 |
| VdbHLH050 | SlbHLH149 |
| VdbHLH050 | SlbHLH158 |
| VdbHLH051 | SlbHLH052 |
| VdbHLH051 | SlbHLH143 |
| VdbHLH052 | SlbHLH089 |
| VdbHLH052 | SlbHLH109 |
| VdbHLH053 | SlbHLH111 |
| VdbHLH053 | SlbHLH112 |
| VdbHLH055 | SlbHLH100 |
| VdbHLH058 | SlbHLH077 |
| VdbHLH058 | SlbHLH098 |
| VdbHLH060 | SlbHLH026 |
| VdbHLH061 | SlbHLH027 |
| VdbHLH062 | SlbHLH107 |
| VdbHLH062 | SlbHLH132 |
| VdbHLH067 | SlbHLH144 |
| VdbHLH067 | SlbHLH145 |
| VdbHLH068 | SlbHLH131 |
| VdbHLH068 | SlbHLH149 |
| VdbHLH068 | SlbHLH158 |
| VdbHLH071 | SlbHLH039 |
| VdbHLH071 | SlbHLH127 |
| VdbHLH073 | SlbHLH033 |
| VdbHLH075 | SlbHLH048 |
| VdbHLH075 | SlbHLH091 |
| VdbHLH077 | SlbHLH072 |
| VdbHLH078 | SlbHLH007 |
| VdbHLH078 | SlbHLH008 |
| VdbHLH079 | SlbHLH010 |
| VdbHLH080 | SlbHLH006 |
| VdbHLH080 | SlbHLH148 |
| VdbHLH081 | SlbHLH012 |
| VdbHLH081 | SlbHLH137 |
| VdbHLH082 | SlbHLH115 |
| VdbHLH082 | SlbHLH124 |
| VdbHLH084 | SlbHLH040 |
| VdbHLH084 | SlbHLH081 |
| VdbHLH084 | SlbHLH105 |
| VdbHLH085 | SlbHLH040 |
| VdbHLH085 | SlbHLH081 |
| VdbHLH085 | SlbHLH105 |
| VdbHLH087 | SlbHLH090 |
| VdbHLH087 | SlbHLH155 |
| VdbHLH088 | SlbHLH046 |
| VdbHLH088 | SlbHLH094 |
| VdbHLH088 | SlbHLH153 |
| VdbHLH089 | SlbHLH090 |
| VdbHLH089 | SlbHLH155 |
| VdbHLH090 | SlbHLH046 |
| VdbHLH090 | SlbHLH094 |
| VdbHLH090 | SlbHLH153 |
| VdbHLH091 | SlbHLH048 |
| VdbHLH091 | SlbHLH091 |
| VdbHLH093 | SlbHLH072 |
| VdbHLH096 | SlbHLH047 |
| VdbHLH097 | SlbHLH006 |
| VdbHLH097 | SlbHLH148 |
| VdbHLH098 | SlbHLH064 |
| VdbHLH099 | SlbHLH034 |
| VdbHLH101 | SlbHLH140 |
| VdbHLH102 | SlbHLH038 |
| VdbHLH102 | SlbHLH049 |
| VdbHLH102 | SlbHLH067 |
| VdbHLH102 | SlbHLH097 |
| VdbHLH102 | SlbHLH122 |
| VdbHLH102 | SlbHLH130 |
| VdbHLH102 | SlbHLH157 |
| VdbHLH103 | SlbHLH002 |
| VdbHLH103 | SlbHLH003 |
| VdbHLH103 | SlbHLH004 |
| VdbHLH103 | SlbHLH018 |
| VdbHLH103 | SlbHLH019 |
| VdbHLH103 | SlbHLH020 |
| VdbHLH103 | SlbHLH119 |
| VdbHLH103 | SlbHLH120 |
| VdbHLH104 | SlbHLH032 |
| VdbHLH104 | SlbHLH117 |
| VdbHLH105 | SlbHLH069 |
| VdbHLH107 | SlbHLH133 |
| VdbHLH108 | SlbHLH107 |
| VdbHLH108 | SlbHLH132 |

**Supplemental Table S6 The paralogous *bHLH* genes in grape, *Arabidopsis* and tomato genome.**

| **bHLH Name1** | **bHLH Name2** |
| --- | --- |
| AtbHLH006 | AtbHLH053 |
| AtbHLH022 | AtbHLH134 |
| AtbHLH022 | AtbHLH135 |
| AtbHLH025 | AtbHLH076 |
| AtbHLH025 | AtbHLH141 |
| AtbHLH029 | AtbHLH106 |
| AtbHLH038 | AtbHLH133 |
| AtbHLH076 | AtbHLH141 |
| AtbHLH125 | AtbHLH148 |
| AtbHLH134 | AtbHLH135 |
| SlbHLH001 | SlbHLH056 |
| SlbHLH001 | SlbHLH075 |
| SlbHLH002 | SlbHLH003 |
| SlbHLH002 | SlbHLH004 |
| SlbHLH002 | SlbHLH018 |
| SlbHLH002 | SlbHLH019 |
| SlbHLH002 | SlbHLH020 |
| SlbHLH002 | SlbHLH119 |
| SlbHLH002 | SlbHLH120 |
| SlbHLH003 | SlbHLH004 |
| SlbHLH003 | SlbHLH018 |
| SlbHLH003 | SlbHLH019 |
| SlbHLH003 | SlbHLH020 |
| SlbHLH003 | SlbHLH119 |
| SlbHLH003 | SlbHLH120 |
| SlbHLH004 | SlbHLH018 |
| SlbHLH004 | SlbHLH019 |
| SlbHLH004 | SlbHLH020 |
| SlbHLH004 | SlbHLH119 |
| SlbHLH004 | SlbHLH120 |
| SlbHLH006 | SlbHLH148 |
| SlbHLH007 | SlbHLH008 |
| SlbHLH012 | SlbHLH137 |
| SlbHLH018 | SlbHLH019 |
| SlbHLH018 | SlbHLH020 |
| SlbHLH018 | SlbHLH119 |
| SlbHLH018 | SlbHLH120 |
| SlbHLH019 | SlbHLH020 |
| SlbHLH019 | SlbHLH119 |
| SlbHLH019 | SlbHLH120 |
| SlbHLH020 | SlbHLH119 |
| SlbHLH020 | SlbHLH120 |
| SlbHLH032 | SlbHLH117 |
| SlbHLH038 | SlbHLH049 |
| SlbHLH038 | SlbHLH067 |
| SlbHLH038 | SlbHLH097 |
| SlbHLH038 | SlbHLH122 |
| SlbHLH038 | SlbHLH130 |
| SlbHLH038 | SlbHLH157 |
| SlbHLH039 | SlbHLH127 |
| SlbHLH040 | SlbHLH081 |
| SlbHLH040 | SlbHLH105 |
| SlbHLH046 | SlbHLH094 |
| SlbHLH046 | SlbHLH153 |
| SlbHLH048 | SlbHLH091 |
| SlbHLH049 | SlbHLH067 |
| SlbHLH049 | SlbHLH097 |
| SlbHLH049 | SlbHLH122 |
| SlbHLH049 | SlbHLH130 |
| SlbHLH049 | SlbHLH157 |
| SlbHLH052 | SlbHLH143 |
| SlbHLH056 | SlbHLH075 |
| SlbHLH067 | SlbHLH097 |
| SlbHLH067 | SlbHLH122 |
| SlbHLH067 | SlbHLH130 |
| SlbHLH067 | SlbHLH157 |
| SlbHLH077 | SlbHLH098 |
| SlbHLH081 | SlbHLH105 |
| SlbHLH089 | SlbHLH109 |
| SlbHLH090 | SlbHLH155 |
| SlbHLH094 | SlbHLH153 |
| SlbHLH097 | SlbHLH122 |
| SlbHLH097 | SlbHLH130 |
| SlbHLH097 | SlbHLH157 |
| SlbHLH107 | SlbHLH132 |
| SlbHLH111 | SlbHLH112 |
| SlbHLH115 | SlbHLH124 |
| SlbHLH119 | SlbHLH120 |
| SlbHLH122 | SlbHLH130 |
| SlbHLH122 | SlbHLH157 |
| SlbHLH130 | SlbHLH157 |
| SlbHLH131 | SlbHLH149 |
| SlbHLH131 | SlbHLH158 |
| SlbHLH144 | SlbHLH145 |
| SlbHLH149 | SlbHLH158 |
| VdbHLH002 | VdbHLH008 |
| VdbHLH002 | VdbHLH029 |
| VdbHLH002 | VdbHLH088 |
| VdbHLH002 | VdbHLH090 |
| VdbHLH003 | VdbHLH015 |
| VdbHLH004 | VdbHLH092 |
| VdbHLH005 | VdbHLH077 |
| VdbHLH005 | VdbHLH093 |
| VdbHLH006 | VdbHLH075 |
| VdbHLH006 | VdbHLH091 |
| VdbHLH008 | VdbHLH029 |
| VdbHLH008 | VdbHLH088 |
| VdbHLH008 | VdbHLH090 |
| VdbHLH011 | VdbHLH087 |
| VdbHLH011 | VdbHLH089 |
| VdbHLH012 | VdbHLH081 |
| VdbHLH013 | VdbHLH037 |
| VdbHLH013 | VdbHLH082 |
| VdbHLH019 | VdbHLH101 |
| VdbHLH021 | VdbHLH105 |
| VdbHLH023 | VdbHLH046 |
| VdbHLH023 | VdbHLH047 |
| VdbHLH023 | VdbHLH104 |
| VdbHLH028 | VdbHLH041 |
| VdbHLH028 | VdbHLH080 |
| VdbHLH028 | VdbHLH097 |
| VdbHLH029 | VdbHLH088 |
| VdbHLH029 | VdbHLH090 |
| VdbHLH031 | VdbHLH052 |
| VdbHLH032 | VdbHLH050 |
| VdbHLH032 | VdbHLH068 |
| VdbHLH037 | VdbHLH082 |
| VdbHLH040 | VdbHLH095 |
| VdbHLH040 | VdbHLH102 |
| VdbHLH041 | VdbHLH070 |
| VdbHLH041 | VdbHLH080 |
| VdbHLH041 | VdbHLH097 |
| VdbHLH042 | VdbHLH043 |
| VdbHLH042 | VdbHLH044 |
| VdbHLH043 | VdbHLH044 |
| VdbHLH046 | VdbHLH047 |
| VdbHLH046 | VdbHLH104 |
| VdbHLH047 | VdbHLH104 |
| VdbHLH049 | VdbHLH067 |
| VdbHLH050 | VdbHLH068 |
| VdbHLH062 | VdbHLH108 |
| VdbHLH070 | VdbHLH097 |
| VdbHLH075 | VdbHLH091 |
| VdbHLH077 | VdbHLH093 |
| VdbHLH080 | VdbHLH097 |
| VdbHLH084 | VdbHLH085 |
| VdbHLH087 | VdbHLH089 |
| VdbHLH088 | VdbHLH090 |
| VdbHLH095 | VdbHLH102 |

**Supplemental Table S7 The number of red spots in 8 days after injection.**

|  | **Number of red spots 8 d after treatment** | **Total number** |
| --- | --- | --- |
| **VC** | 14 | 100 |
| **OE** | 34 | 100 |

**Table S8 The number of coloring fruits in 16 days after injection.**

|  | **Number of coloured fruits 16 d after treatment** | **Number of decayed fruits** | **Total number** |
| --- | --- | --- | --- |
| **VC** | 79 | 21 | 100 |
| **OE** | 83 | 17 | 100 |

**Supplemental Table S9 List of primers used for RT-PCR, qRT-PCR and construction of recombinant plasmid.**

| **Gene name** | **Gene identifier** | **Forward primer (5’-3’)** | **Reverse primer (5’-3’)** |
| --- | --- | --- | --- |
| *AtCHI* | AT3G55120.1 | CTCCTCCAATCCATTATTCCTCG | TTTCCCTTCCACTTGACAGATAGAG |
| *AtCHS* | AT5G13930.1 | GCATCTTGGCTATTGGCACTG | CGTTTCCGAATTGTCGACTTGT |
| *AtF3H* | AT3G51240.1 | GTGTTTAGCGACGAAATCCCG | ACGAGCGAGACGAGTCATATCC |
| *AtDFR* | AT5G42800.1 | CAAACGCCAAGACGCTACTCA | CATTCACTGTCGGCTTTATCACTTC |
| *AtLDOX* | AT4G22880.1 | ACGGTCCTCAAGTTCCCACAA | CAGCTCCTCAATACAATTCTCACG |
| *AtUGT78D2* | AT5G17050.1 | ACCGCACAATCCAACTCTTCG | TCCTGTGGTCTCCCGCTAAA |
| *AtACT2* | AT3G18780.1 | AACCACTATGTTCTCAGGTATCGCT | TGGACCTGCCTCATCATACTCG |
| *VdbHLH003* | VIT_01s0127g00860 | TGCAGCAATTCAGTTACACC | TTTTCCAGTCCTCCTTCGGTA |
| *VdbHLH004* | VIT_01s0026g01140 | AGATGAGAACCGTGATTCCAA | AGAATTTTCATCCTCTCGCTGA |
| *VdbHLH033* | VIT_06s0061g00720 | ATCCTGATTCGGTTATCGACT | GAACGTAACGCATAGAGCTTC |
| *VdbHLH037* | VIT_07s0104g00090 | ACACCAAGTACCGCGACGAGA | TCCGAGAATCGAAGCCTTGTCC |
| *VdbHLH062* | VIT_12s0059g02650 | AAAAGAACTTGGACCCCGAAA | AGCTTCTGTGCTTCACTTCGT |
| *VdbHLH097* | VIT_18s0001g06650 | CAGTACTCAACTCGTGCAAC | ACACTGAGTGAATATAGCACCA |
| VdActin | [VIT_04s0044g00580](http://plants.ensembl.org/Vitis_vinifera/Gene/Summary?g=VIT_04s0044g00580;r=4:21427866-21431057;t=VIT_04s0044g00580.t01;db=core) | GTGCCTGCCATGTATGTTGCC | GTCACGTCCAGCAAGGTCAAG |
| pHB-Hyg | XM_003071606 | TCGTTATGTTTATCGGCACTTTG | GCGTCTGCTGCTCCATACAAG |
| VlActin | VIT_204s0044g00580 | CTTGCATCCCTCAGCACCTT | TCCTGTGGACAATGGATGGA |
| VdbHLH037-3301 | VIT_07s0104g00090 | GAACACGGGGGACTCTTGACATGGCTGCGCCGCCGAATAGC | TAGAAATTTACCCTCAGATCTCAGCACTGGGGTATTATTTG |
| VdbHLH037-pHB | VIT_07s0104g00090 | CTCTTCTCAAGCTTGGATCCATGGCTGCGCCGCCGAATAGC | CTCCTGCAGCTCGAGGATCCTCAGCACTGGGGTATTATTTG |
| AtUBQ3 | AT5G03240 | CGGAAAGACCATTACTCTGGA | CAAGTGTGCGACCATCCTCAA |

**The original image**

Image 1


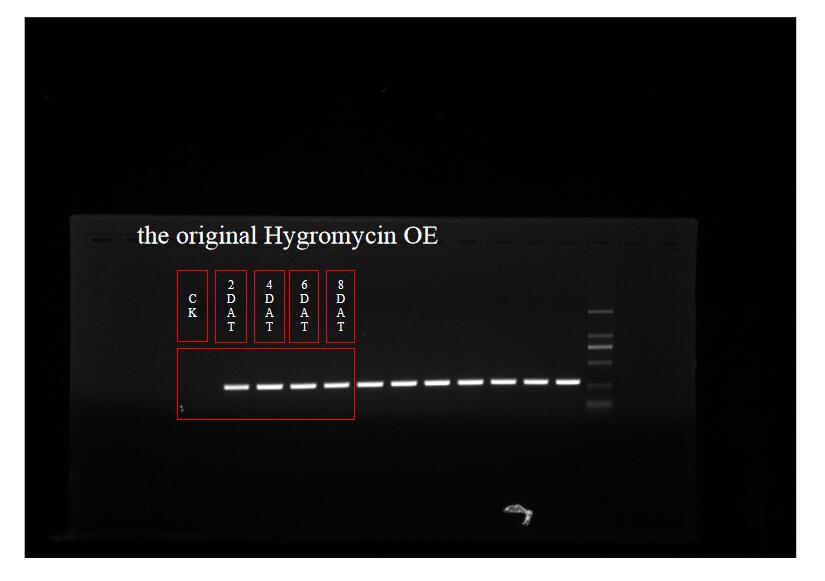


Image 2


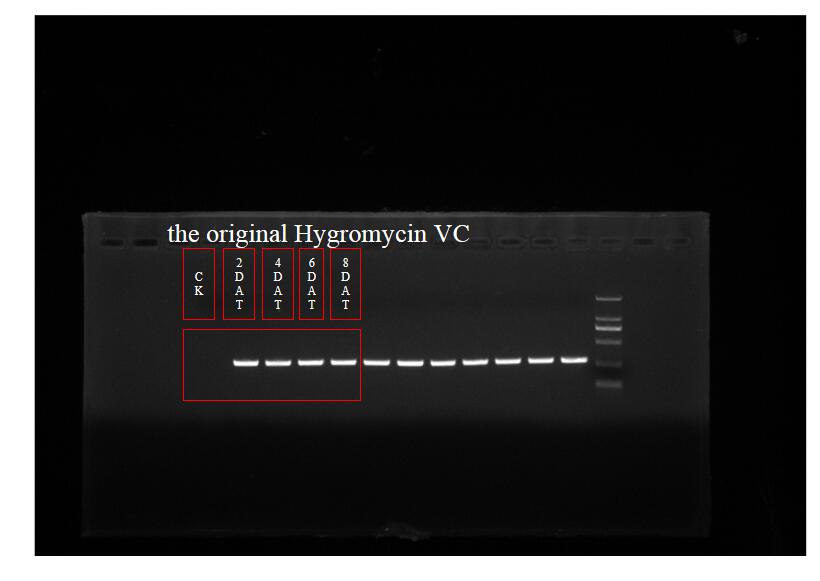


Image 3


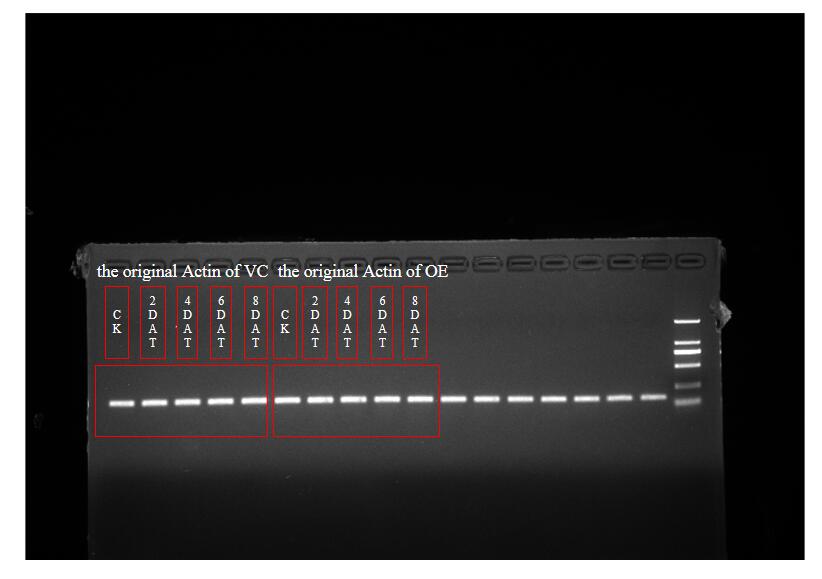

Supplement: Supplementary file 1 — Supplementary Information [file 41598_2021_85754_MOESM1_ESM.doc]
